# Reconstructing healthcare networks from patient transfer data: A systematic review

**DOI:** 10.1371/journal.pdig.0001718

**Published:** 2026-09-15

**Authors:** Elise Hodbert, Pascal Crépey, Pauline Clin, Victor Renault, Gabriel Birgand, Laura Temime

**Affiliations:** 1 Cibles et Médicaments des Infections et de l’Immunité – UR 1155, Nantes Université, Nantes, France; 2 Laboratoire Modélisation, épidémiologie et surveillance des risques sanitaires, Conservatoire national des arts et métiers, Paris, France; 3 RSMS – Inserm U 1309, Arènes - UMR 6051, EHESP, CNRS, IEP Rennes, Université de Rennes, Rennes, France; 4 Population Genetics, Department of Life Science Systems, School of Life Sciences, Technical University of Munich, Freising, Germany; 5 Centre d’Appui à la Prévention des Infections Associées aux Soins des Pays de la Loire, Centre Hospitalier Universitaire (CHU) - Le Tourville, Nantes, France; 6 National Institute for Health Research Health Protection Research Unit in Healthcare Associated Infections and Antimicrobial Resistance at Imperial College London, London, United Kingdom; 7 Unité PACRI, Institut Pasteur, Conservatoire national des arts et métiers, Paris, France; Liverpool John Moores University - City Campus: Liverpool John Moores University, UNITED KINGDOM OF GREAT BRITAIN AND NORTHERN IRELAND

## Abstract

Healthcare facilities are interconnected via patient transfers, facilitating pathogen spread. We systematically reviewed studies reconstructing patient-sharing networks. Following PRISMA 2020 guidelines and a preregistered protocol (CRD42024559127), we searched PubMed, Scopus and IEEE Xplore up to October 1, 2025. We included articles presenting networks of at least three healthcare facilities or units connected by observed patient transfers, excluding those restricted to specific patient groups. Seventy-nine articles were included from 5,721 screened, describing 50 distinct networks published across multiple disciplines. Overall confidence in study findings, assessed through a modified QuADS tool, was good (mean total score of 21/33), although precision in the description of underlying patient transfer data was often limited. Most networks were reconstructed in Europe and North America. The majority were restricted to hospitals, with nursing homes and other healthcare facilities rarely integrated, and 10 (20%) used temporal network approaches. While most articles reported descriptive statistics and network metrics, they focused mainly on density and degree (and its variants), with other metrics only rarely calculated. Only 27 (35%) articles developed mathematical models of pathogen transmission, including 12 that simulated public health interventions. Overall, this review highlights the value of patient-sharing networks to characterize healthcare connectivity and understand pathogen spread. Clearly articulating the methodological assumptions underlying network reconstruction and tailoring them to specific research questions is key to fully leveraging the reconstructed networks for public health purposes. In particular, including a broader range of facility types and more temporal modelling can help develop more realistic models of pathogen spread over these networks. This review was funded by the French National Research Agency (ANR 22-PAMR-0003).

## Author summary

Healthcare systems are strongly connected through patient movements between care facilities. While these transfers are essential for patient care, they can also facilitate the spread of pathogens across healthcare settings. In recent years, digital patient transfer data have been used to map healthcare networks, with the aim of better understanding care organization and infection risks. However, no review has examined all studies reconstructing patient-sharing networks between healthcare facilities. In this study, we review 79 research articles that built such networks using routine patient transfer data. Together, these studies described 50 distinct networks from multiple countries, mainly in Europe and North America. We find that most networks focused on hospitals only, overlooking other facilities such as nursing homes and rehabilitation centers. Moreover, most studies relied on static representations that fail to capture the temporal dynamics of patient movements. About a third of the studies also developed a mathematical model of pathogen spread on these networks, enabling simulations to assess infection control strategies. Overall, our findings show that patient transfer networks are a promising yet underused digital tool for understanding healthcare systems. Expanding their scope and making better use of temporal data could strengthen surveillance and support more effective infection prevention.

## Introduction

Inter-institutional patient transfers constitute a key process for healthcare delivery [1]. In the United States, 1.4 million patients were transferred between hospitals in 2009, which means that about 4% of all hospital admissions involved a transfer [2]. In addition to inter-hospital transfers, patient transfers also frequently occur between units within facilities [3]. These movements contribute to the spread of pathogens [4–9]. It is therefore necessary to characterize patient transfer patterns, to better understand the spread of communicable diseases in healthcare settings and recommend control strategies.

Social network analysis offers a powerful framework to analyze these systems. It conceptualizes systems as networks composed of nodes connected by edges, enabling visualization and analysis of relationships within the healthcare system. Over the past 15 years, this framework has been used to reconstruct patient-sharing networks in several countries, including the Netherlands, the United Kingdom, the United States, and France [8,10–16]. By analyzing the networks’ properties and developing mathematical models of pathogen spread over them, these studies proposed innovative surveillance and control strategies, notably to target antibiotic-resistant bacteria. Previous reviews addressed some aspects of patient-sharing networks. Assab et al. (2017) compiled studies that developed mathematical models of disease transmission across hospital networks [17]. DuGoff et al. (2018) reviewed all networks based on shared patient care among physicians, focusing on theoretical frameworks and network reconstruction methods [18]. More recently, Korsberg et al. (2024) expanded this concept by analyzing studies that quantified relationships among healthcare providers, which could be either individual clinicians or facilities [19].

However, to our knowledge, no systematic review has specifically examined studies reconstructing networks in which nodes are healthcare facilities or units and edges are patient transfers. This systematic review aims to identify the studies reconstructing such networks, summarize the methodological assumptions underlying network reconstruction, characterize the resulting networks, and describe the network-based analyses, particularly the propagation models developed on the networks.

## Methods

We conducted a systematic review following the PRISMA 2020 guidelines [20]. The PRISMA checklist is provided in S1 File. The study protocol was preregistered on June 28, 2024 in PROSPERO (CRD42024559127).

### Search strategy and selection criteria

We searched PubMed, Scopus and Institute of Electrical and Electronics Engineers (IEEE) Xplore Digital Library for articles published from database inception to October 1, 2025. The search query included three mandatory terms referring to (i) healthcare facilities (e.g., “hospital” or “nursing home”), (ii) patient movements (e.g., “patient transfer” or “patient sharing”), and (iii) the concept of a group of facilities (e.g., “network” or “interhospital”). A fourth term was used to exclude terms that were outside the scope of this review. The detailed search query is provided in S1 Table.

### Screening

All results from the search query were independently screened by two reviewers (EH and VR) for eligibility based on the inclusion criteria, using the Covidence systematic review software (Veritas Health Innovation, Melbourne, Australia). Any conflicts were resolved by two other investigators (LT and GB).

Screening was first conducted based on titles and abstracts, followed by a full-text assessment. We included only original research articles and excluded reviews, including Cochrane reviews and meta-analyses. We included articles that met the following criteria: (i) used observed data on patient or resident movement, (ii) presented a network constructed from these patient transfer data, (iii) had network nodes representing healthcare facilities or units and edges representing patient transfer, and (iv) presented a network with at least three nodes. We excluded articles that selected patients based on a specific pathology.

### Data extraction

For all included articles, one author (EH) extracted information on the following characteristics: year of publication, journal discipline, network scale, location, patient transfer database and duration. To assess network reconstruction assumptions, we reported patient movement scale (intrafacility, interfacility, or both), types of facilities included, maximum delay between two consecutive hospital stays to be considered as a patient transfer, and network temporality (static or temporal). To evaluate network size, we extracted the number of facilities, number of patient transfers, and the descriptive metrics reported. When disease transmission models were developed, we additionally collected information on the type of model used, the pathogen studied, the interventions simulated, and the epidemiological data integrated. When information was not reported in an included article, descriptive statistics were conducted using only articles with available data for the variable considered.

Because a given network could be described in multiple articles, information was extracted separately each time; similarly, when an article reported more than one network, information was extracted for each network.

Network metrics were classified using an adaptation of the framework proposed by Korsberg et al., 2024 [19]: (i) node-level measures, (ii) dyad-level measures, (iii) triad-level measures, and (iv) network-level measures. Node-level measures quantify the position and relationships of individual healthcare facilities or units within a network. Dyad-level measures describe the connections between pairs of facilities or units, while triad-level measures assess relationships among groups of three facilities or units. Network-level measures provide an overall view of the system by summarizing the relationships and positions of all nodes in the network.

### Definitions

In this review, a *network* refers to the structure reconstructed from a specific patient transfer database by a given research team. We considered that a study presented a network if it provided a matrix of transitions between facilities or units, descriptive statistics or a visual representation of the network. An article could describe multiple networks; in such cases, each network was reported separately in this review. Conversely, a given network could be featured in multiple scientific articles, sometimes with minor differences, for instance in the number or type of facilities included.

We distinguished two types of transfers based on the maximum time interval allowed between hospital stays. *Direct transfers* referred to consecutive admissions occurring within a 48-hour time frame, while *indirect transfers* permitted an intermediate stay in the community between two hospitalizations. We classified patient transfers as *intrafacility* when they occurred between units within the same facility or group of facilities, *interfacility* when they occurred between different facilities, and *intrafacility and interfacility* for articles that considered inter-unit movements across multiple facilities. We classified networks as *temporal* when multiple networks were reconstructed at successive time intervals, less than or equal to three months, and *static* otherwise.

### Confidence assessment

The guidelines of the Quality Assessment for Diverse Studies (QuADS) tool used for reporting quality in systematic reviews of mixed- or multi-method studies were adapted to assess the quality and methodological biases of included articles [21]. We removed criteria that were not applicable to our review, resulting in a final set of 11 criteria. Articles were rated from 0 to 3 for each criterion (S2 Table).

Ethics statement: According to the French law, no ethical review was needed to conduct this study.

## Results

### Descriptive findings

**Fig 1:**
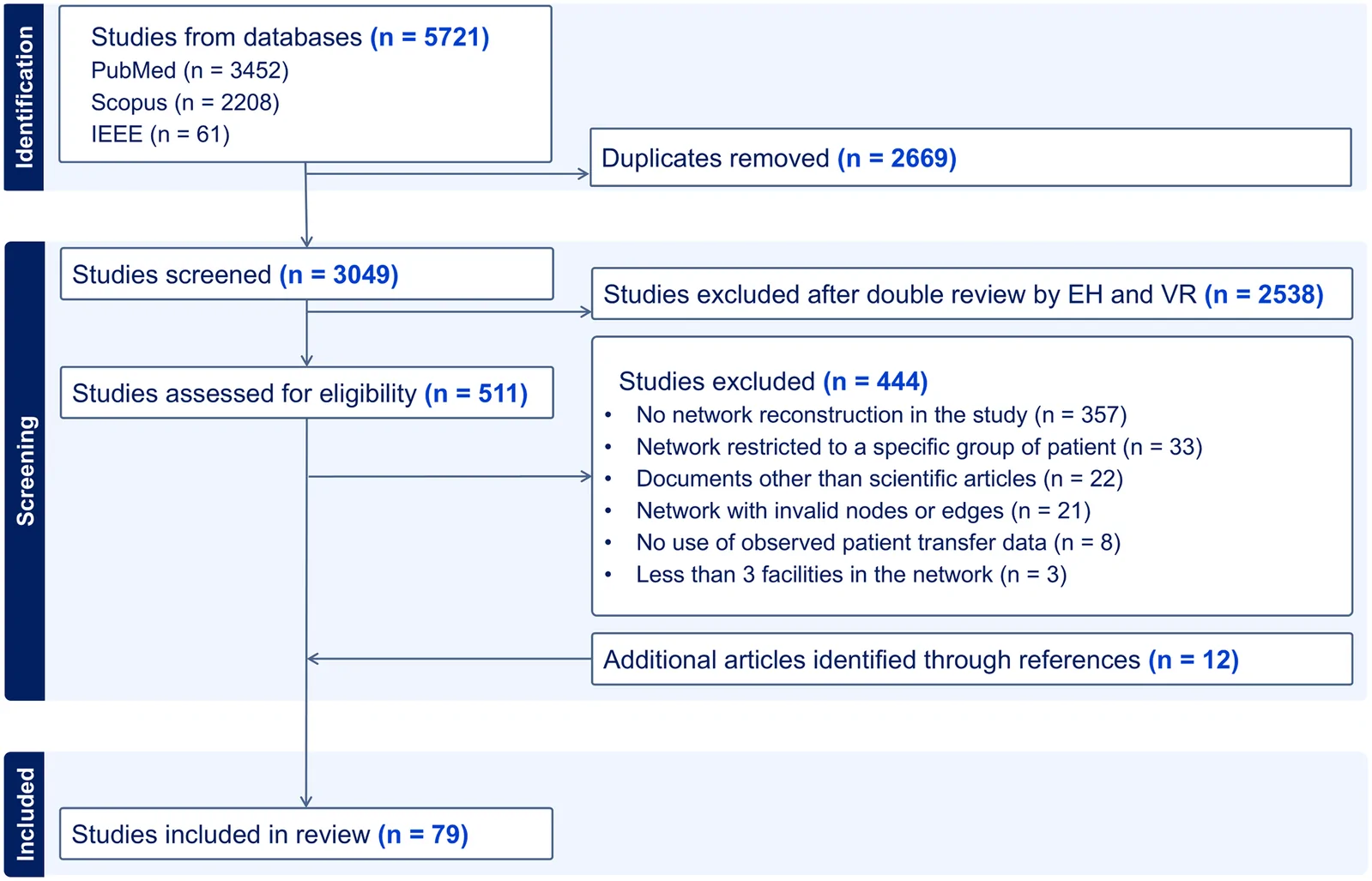
Flow diagram of study selection.

We found 5,721 articles across PubMed, Scopus and IEEE, of which 2,669 duplicates were removed (Fig 1). Of the 3,049 abstracts assessed, 511 were selected for full-text screening. Among them, 444 articles were excluded, mainly due to the absence of network reconstruction (n = 357). To identify any additional relevant articles, we examined the references of all included articles, leading to the identification of 12 additional articles meeting inclusion criteria. In total, 79 articles were included in the review [4–10,12–16,22–85]. Inter-rater agreement was substantial, with a Cohen’s kappa of 0.74 (S3 Table). The full list of articles assessed at the full-text screening stage, including excluded articles and reasons for exclusion, is provided in S4 Table. S5 Table presents all data extracted from the included articles.

**Fig 2:**
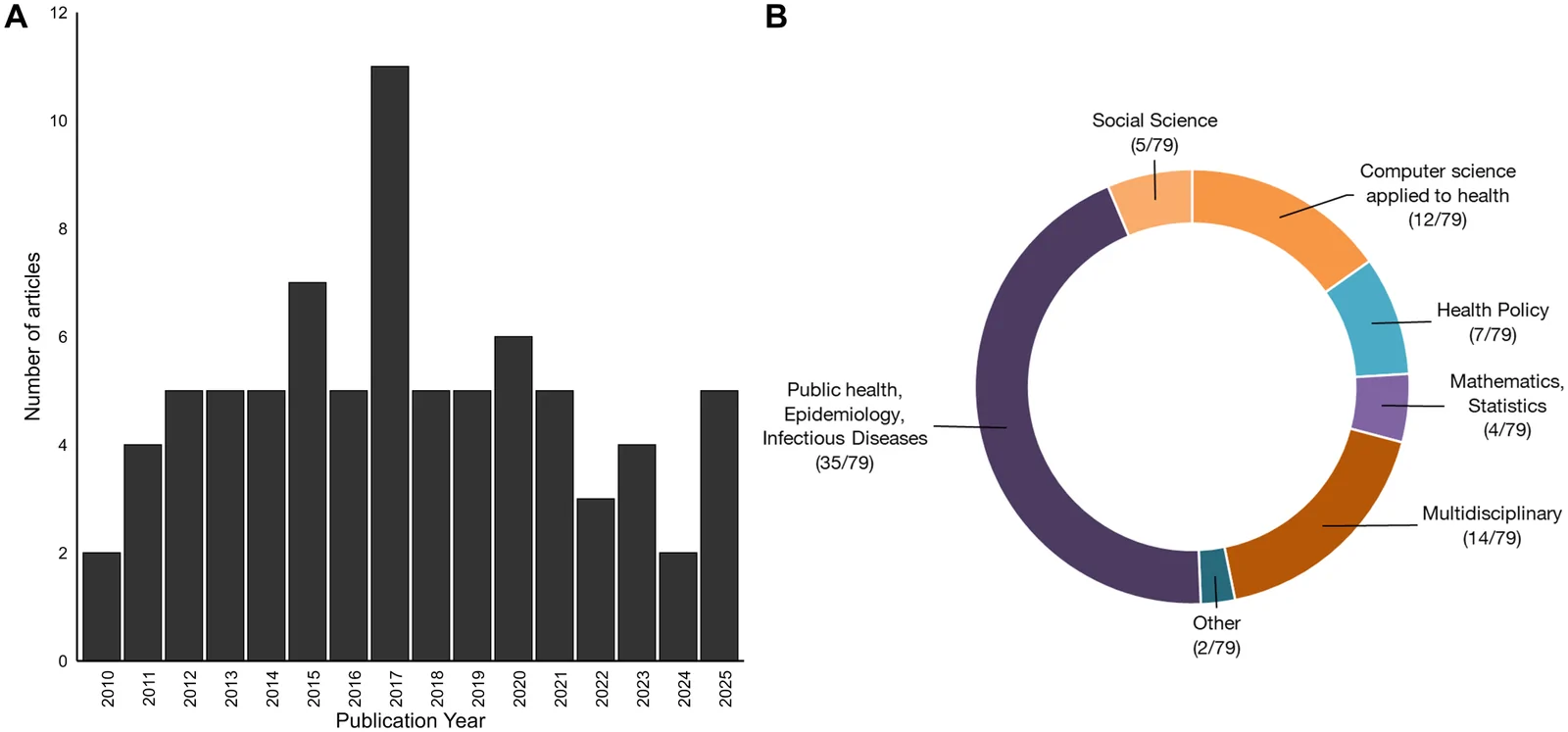
Characteristics of included articles. **(A)** Annual publication counts **(B)** Distribution across journal categories.

The included articles were published from 2010 to 2025, with the highest publication frequency occurring in 2017 (Fig 2A). They were published in journals spanning multiple academic disciplines, most frequently in *Public health, epidemiology, and infectious diseases* journals, which accounted for 35/79 articles (Fig 2B).

### Most networks were reconstructed in Europe and North America

**Fig 3:**
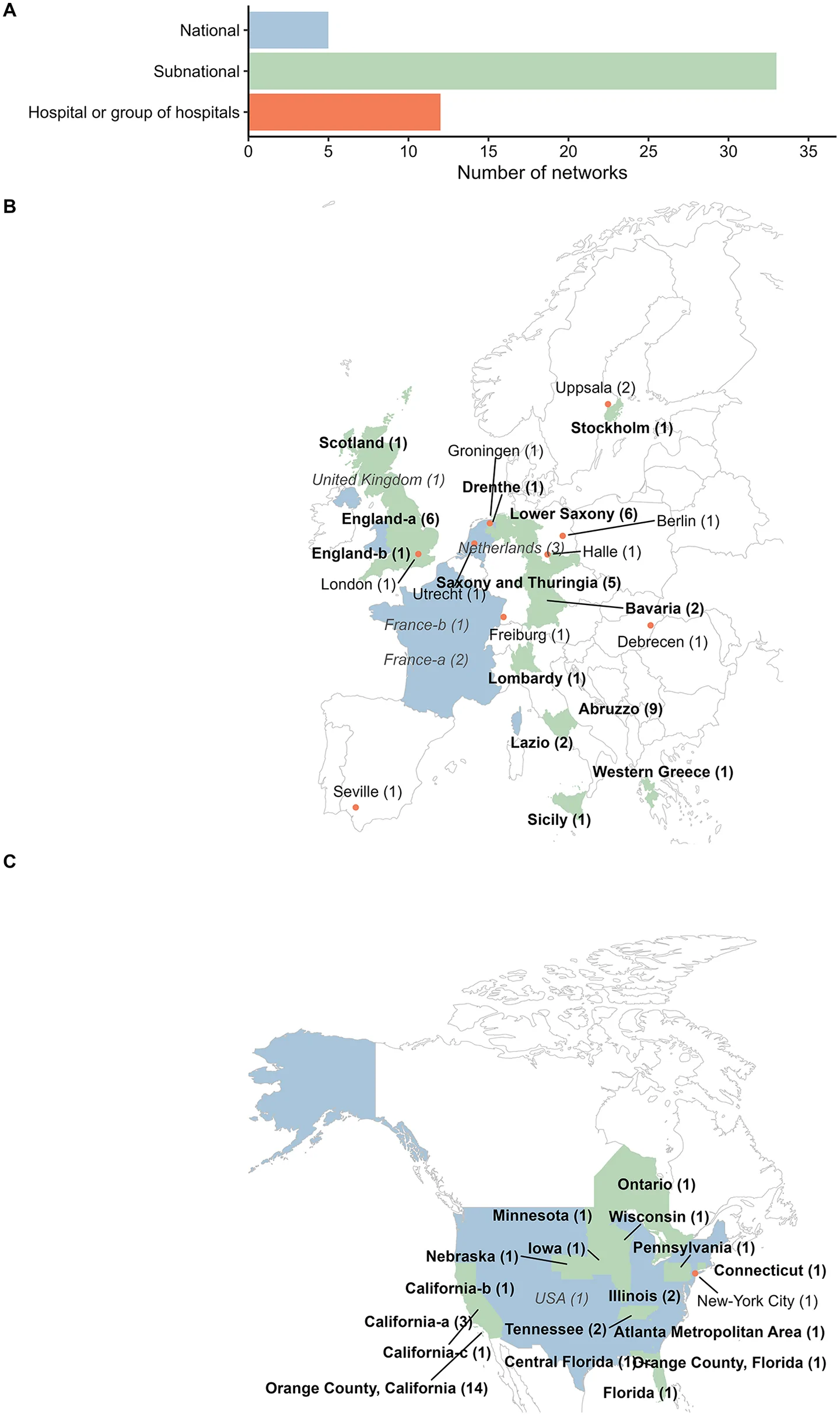
Scale and geographic distribution of the 50 networks. **(A)** Number of networks by scale **(B)** European networks **(C)** North American networks. Numbers in brackets indicate how many articles reported each network if more than one. Figure created using the Natural Earth base layer from www.naturalearthdata.com under the PDDL license.

The 79 included articles presented 50 unique networks (S6 Table). Among them, 7/79 (8.9%) articles presented two or more networks [11,22–28], and 14/50 (28.0%) networks were presented in two or more articles (mean number of articles per network 2, range 1–14). Regarding network scale, 5/50 (10.0%) were national, 33/50 (66.0%) were subnational, and 12/50 (24.0%) comprised only individual hospitals or groups of hospitals (Fig 3A). Most networks were reconstructed in Europe (26/50, 52.0%) (Fig 3B) and North America (19/50, 38.0%) (Fig 3C), while a small number of networks were reconstructed in Asia (3/50, 6.0%) and Oceania (2/50, 4.0%).

### Data sources for network reconstruction

**Fig 4:**
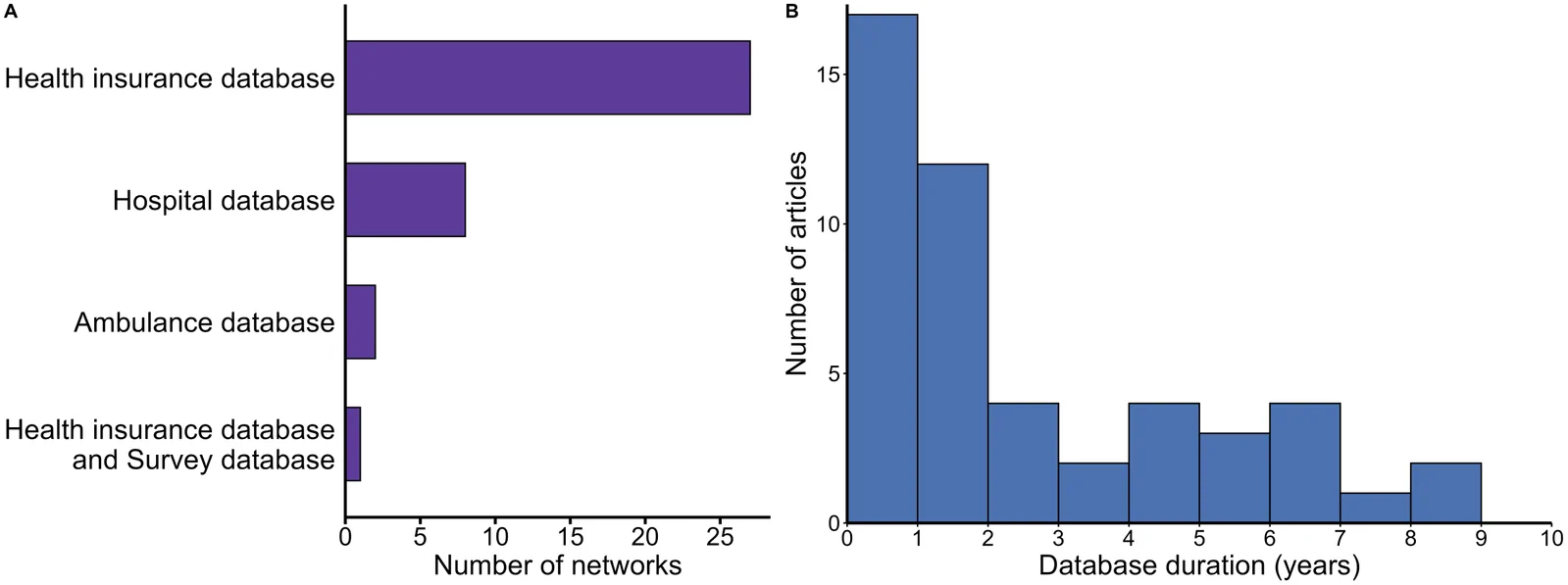
Types and duration of databases in the networks. **(A)** Frequency of database types. **(B)** Distribution of database duration.

Among the 50 networks, 27 (54.0%) relied on health insurance databases, most of which were public systems, including Programme de médicalisation des systèmes d’information (PMSI) in France and Medicare in the United States [16,29] (Fig 4A), and one network was reconstructed with a combination of health insurance and survey databases [29]. Database durations ranged from 3 months to 9 years, with a median duration of 2 years (Fig 4B). S1 Fig presents the types and durations of the databases used in the 79 articles separately, showing similar results.

### Most networks included only hospitals and were static rather than temporal

**Fig 5:**
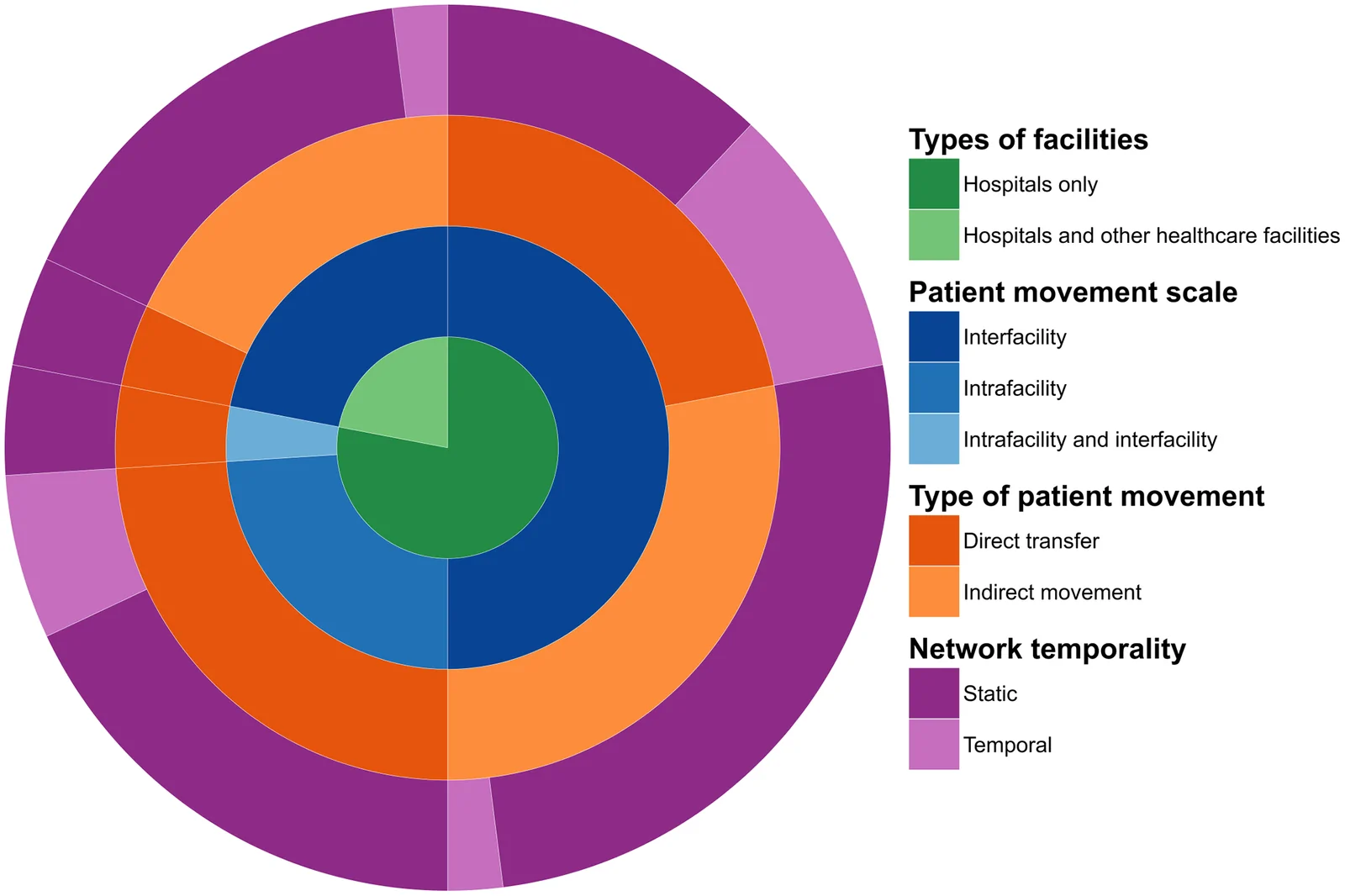
Methodological choices for network reconstruction. Networks were described as including “Hospital and other healthcare facilities” when they were presented as including facilities other than hospitals in at least one article. Patient transfers were labeled “Indirect” when at least one article presenting the network allowed for a stay in the community between two hospital stays. Networks were considered “Temporal” when a temporal reconstruction was used in at least one article.

Most networks considered exclusively hospitals (39/50, 78.0%), excluding other types of healthcare facilities such as nursing homes or rehabilitation centres (Fig 5). Most articles focused on interfacility transfers (36/50, 72.0%), while 12/50 (24.0%) considered intrafacility movements and 2/50 (4.0%) combined both. 27/50 (54.0%) networks included only direct transfers, while 23/50 (46.0%) networks included indirect transfers. The maximum interval allowed between two consecutive stays varied between 10 days and 8 years, with 6/50 (12.0%) networks allowing for more than a year between two hospital stays (S7 Table). Most articles reconstructed a single static network (40/50, 80.0%), regardless of the type of movement or scale considered. All articles that focused exclusively on intrafacility movements considered only direct transfers. All articles that incorporated facilities beyond single hospitals or hospital groups addressed interfacility or mixed movements.

### Network facility counts varied strongly across scales

**Fig 6:**
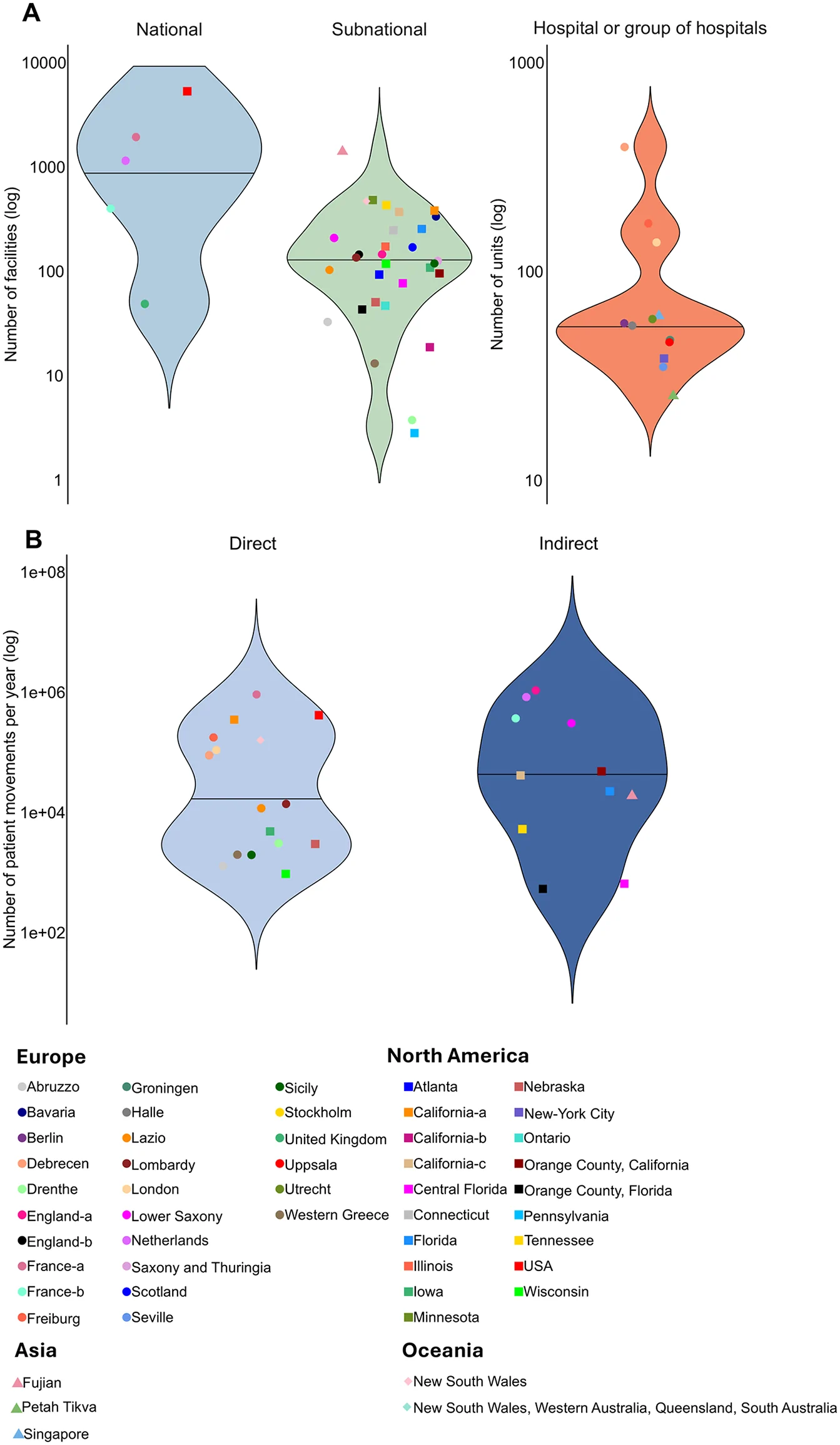
Network characteristics. **(A)** Number of facilities **(B)** Number of patient transfers. For networks described in multiple articles, the highest reported value is shown.

National networks included a median of 826 facilities (2.5–97.5% quantiles: 60–5,036), subnational networks 116 facilities (12–504), and hospital-level networks 58 units (29–340) (Fig 6A). 2/79 articles did not report the number of facilities included in their network [30,31]. S2 Fig presents the network characteristics for each article separately and shows that the facility counts were generally consistent across articles presenting a given network.

### Patient transfer counts varied according to transfer type

The median number of patient transfers was 14,347 (2.5–97.5% quantiles: 1,205–821,293) when considering only direct transfers and 46,659 (634–1,140,436) when indirect transfers were included (Fig 6B). Patient transfer counts were not reported in 46/79 (58.2%) articles. Among them, some described their database using alternative measures such as the total number of unique patients [26–28,30–36] or the total number of admissions to healthcare facilities [24,26,29,30,33,35,37,38].

### Network descriptive metrics

**Fig 7:**
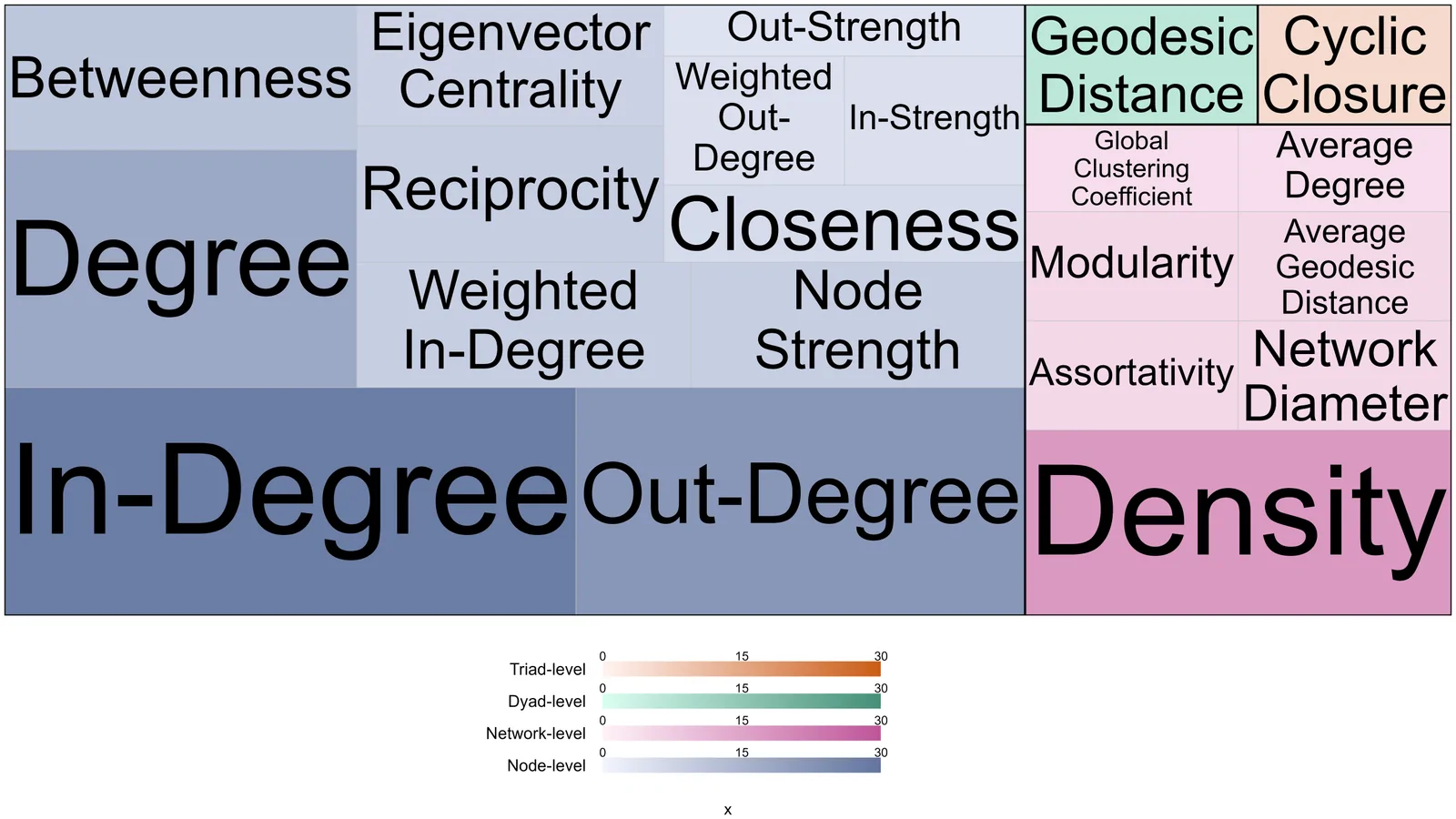
Reported network metrics in the included articles, categorized by analytical level (node-, dyad-, triad-, and network-level measures). Color gradients indicate the number of occurrences.

Overall, 59/79 (74.7%) articles used at least one metric to describe their network (Fig 7). The most used metrics were density, degree and its variations including in-degree and out-degree. In addition, 13/79 (16.4%) used a community detection method to identify clusters within their network. For greater detail, S8 Table lists all metrics reported in the included articles.

### Epidemiological use of the networks

**Fig 8:**
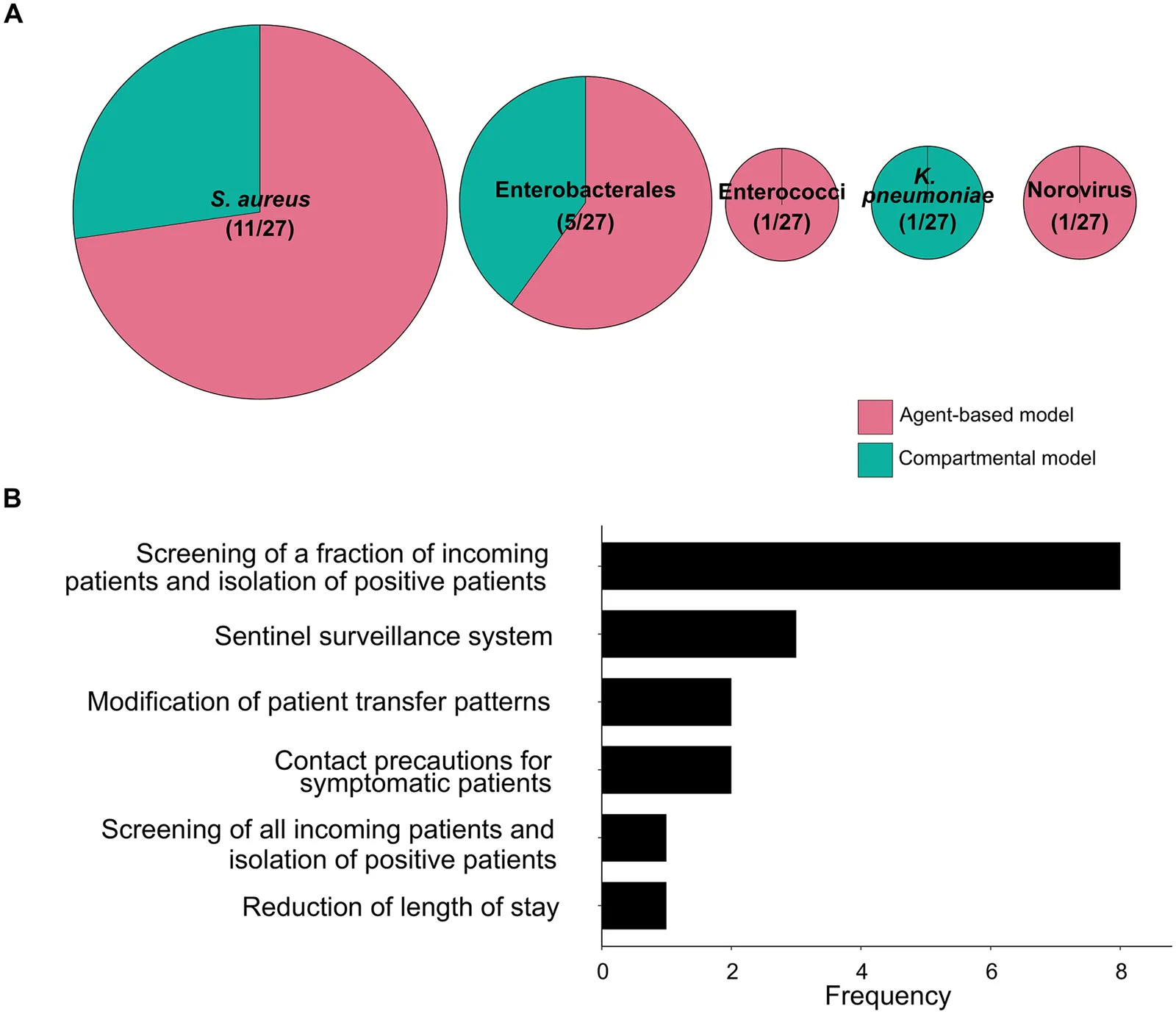
Interventions simulated in the included articles.

Overall, 27/79 (34.2%) articles developed a mathematical model of pathogen transmission. 23/27 (85.2%) models focused on a specific pathogen, most frequently *S. aureus* (11/27, 40.7%) and Enterobacterales (5/27, 18.5%) (Fig 8A). 17/27 (63.0%) models were agent-based, and 10/27 (37.0%) models were compartmental. 12/27 (44.4%) models integrated epidemiological data, and 12/27 (44.4%) articles simulated at least one public health intervention (Fig 8B).

Most modeling studies concluded that pathogen spread was strongly shaped by facilities’ position within the network, with highly connected hospitals, long-term acute-care hospitals and nursing homes acting as strong contributors to regional dissemination or persistence [5,9,10,26,29,36,39,40]. While multiple studies showed that high network connectivity facilitated spatial dissemination [5,9,10,26,28,29,39–42,86], some models suggested that increased inter-hospital transfers could dilute local prevalence, depending notably on network structure [24, 28].

Importantly, several studies indicated that modeling outcomes were sensitive to network reconstruction methodology. Models including only direct transfers underestimated both the number of facilities reached and the predicted system-wide prevalence [6,7,29,43]. In addition, using static networks could bias the model by assuming that all links were simultaneously active, whereas temporal networks preserve the order and timing of patient movements and therefore better capture transmission chains [43].

Among studies that simulated health interventions, 8/27 (29.6%) articles simulated the screening of a fraction of incoming patients with isolation of positives, generally suggesting potential reductions in pathogen prevalence [6,24,26,38,39,42,43]; for instance, screening 30–40% of patients could potentially halve norovirus prevalence within 100 days [86]. 3/27 (11.1%) articles simulated a sentinel surveillance system [11,36,44], and indicated that a small number of sentinel hospitals might be sufficient to shorten detection times [11] and effectively monitor *S. aureus* and Enterobacterales spread [36,44]. Reducing the lengths of stay and readmission rates seemed to lower pathogen prevalence [24,38]. The impact of contact precautions was pathogen-specific, reducing norovirus transmission [86] but showing minimal effects on MRSA [38]. Overall, coordinated interventions generally outperformed facility-level interventions [26,36,39,42,45].

### Confidence assessment

**Fig 9:**
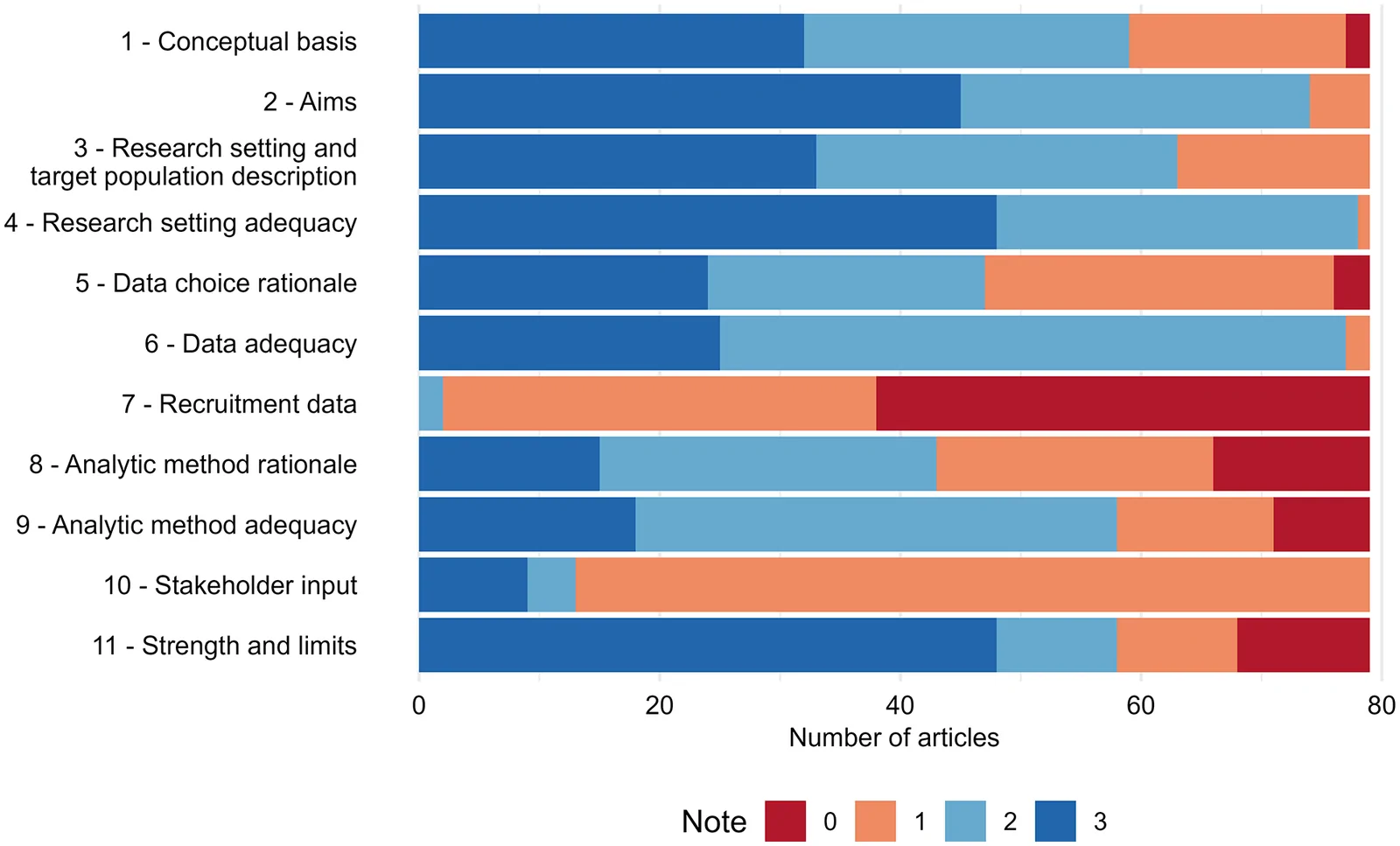
Confidence assessment criteria of included articles adapted from Harrison et al., 2021 [21].

Overall, the articles were based on a clear conceptual basis, with well-defined aims, thorough descriptions of the research setting, good adequacy of the research setting in relation to the stated aims, and a good description of strengths and limits (Fig 9). Although the rationale for selecting the patient transfer databases was not always clearly articulated (32/79, 40.5% with a rating of 0 or 1), the adequacy of the data sources was generally good. The criterion most frequently unmet by the articles was the accessibility of the underlying recruitment data: most articles either used descriptive statistics (36/79, 45.6% with a rating of 0) or did not describe their patient transfer database (41/79, 51.9% with a rating of 0), which limited the understanding of these datasets. Although the methods used to analyze the network were generally adequate, some articles did not justify their methodological choices (36/79, 45.6% with a rating of 0 or 1). Most articles demonstrated insufficient stakeholder involvement in the design and analysis processes (66/79, 83.5% with a rating of 1). The scores for each included article are detailed in S9 Table. S3 Fig presents the confidence assessment stratified by publication period. Scores for criterion #7 (“Recruitment data”) increased from 0.33 in 2010–2014 to 0.55 in 2015–2019 and 0.60 in 2020–2025, suggesting that in this regard, study quality improved over time.

## Discussion

### Review findings

Our systematic review highlights the value of social network analysis for investigating patient pathways. The included articles were published in journals from diverse fields, including public health, social sciences and mathematics. This interdisciplinarity can provide multiple perspectives that enhance our understanding of patient-sharing networks. Additionally, most networks were reconstructed in Europe and North America, while other continents were underrepresented. Few networks included types of facilities other than acute care hospitals, including nursing homes and rehabilitation centres, and few networks were temporal. The network-based analyses varied substantially between the articles, including descriptive statistics, descriptive metrics, and mathematical models of pathogen transmission. Overall, the confidence assessment was good across the included studies, although the description of the underlying patient transfer data was limited in multiple studies.

### Implications for future work

This review raised several points that can inform future works.

First, most networks were reconstructed in Europe or North America, which limits our understanding of patient-sharing networks in other regions of the world. This geographic imbalance may partly reflect unequal access to patient transfer databases. In low-resource settings, very few large-scale patient-stay databases are available [31,46–48], whereas in Europe and North America, large administrative datasets have enabled the reconstruction of comprehensive healthcare facility networks [14]. Bridging this gap will require investments in digital health infrastructure in LMICs, including designing routine recording of electronic health records with admission and discharge data, and secure governance frameworks for data linkage and sharing. These developments are important from an equity perspective, as they would help extend network-based surveillance and modelling approaches to currently underrepresented settings. Even within Europe, substantial differences exist between countries. For example, in Germany, around 10% of the population is privately insured and 90% of the population is insured through the public health insurance system, which is regionally fragmented due to the country’s federal structure. This fragmentation limits the possibility of reconstructing a comprehensive national network, as reconstructions of region-specific networks would miss cross-border patient transfers [6,7,24,26–28,35,43].

Second, the quality and comprehensiveness of patient transfer databases varied substantially across articles. Several articles acknowledged that their databases were not exhaustive [31,37,49], which can compromise network quality, even if certain analytic approaches may mitigate this issue [27]. The extent of missing data depends on the type of database used for network reconstruction. Di Vincenzo (2018) suggested that administrative databases are more precise, detailed, and complete than survey-based databases [50]; these sources should therefore be prioritized whenever accessible.

Third, regarding methodological choices in network reconstruction, most networks focused exclusively on hospitals, excluding other types of healthcare facilities. However, these institutions are strongly interconnected with hospitals and can act as important pathogen reservoirs, which makes their inclusion particularly relevant in articles modelling pathogen transmission. Notably, nursing homes have been identified as major reservoirs of antibiotic-resistant pathogens [8,12,29,39,51–54]. When such data are available, they should therefore be integrated into the network. In addition, most articles considered only interfacility transfers, while patterns of patient transfers within facilities are also important to study, as they can strongly affect hospital performance and contribute to increased infection risk [26,30,55–57, 87]. Some articles did not include indirect patient transfers, despite the fact that these can represent up to 94–99% of all transfers depending on the allowed interval between two hospital stays [6,7,43,88], and can significantly increase pathogen prevalence [7]. Decisions about whether to include indirect transfers, and the maximal allowed duration between two stays, therefore have a major impact on the resulting network structure and predicted pathogen spread across healthcare facilities. They should be informed by the natural history of infection of the pathogen considered, particularly the mean time to recovery. While short-lived infections, such as influenza, may justify focusing on direct transfers or indirect transfers with a short maximal time between two consecutive stays, pathogens able to persist for months in colonized individuals, such as MRSA, should account for indirect transfers over longer time windows. Moreover, most articles reconstructed a single static network over the full period available in their database. Temporal networks capture patient transfer fluctuations over time, including weekly or seasonal variations, such as higher patient flows in winter due to respiratory viruses [89]. They may be more appropriate for studies aiming to model pathogen transmission, since purely static networks may incorrectly estimate transmission risks by assuming all transfer links exist at all times [43]. However, finer temporal resolutions rely on scarser patient transfer data, which may reduce the stability of network estimates. Sliding time windows can partly address this issue by increasing the number of observations, and can also smooth daily fluctuations. Conversely, static networks may provide a global overview of patient flows when temporal analyses show stable patient-sharing patterns, as reported in some studies [16,50]. The choice between static and temporal networks should therefore be guided by the research question, data resolution, and temporal stability of patient flows. For studies including indirect transfers, the temporal scale used to reconstruct the network should also be longer than the maximal allowed interval between two stays, to avoid assigning indirect transfers across incompatible time windows. Furthermore, studies reconstructing temporal networks should explicitly consider structural changes in the healthcare system, such as temporary ward closures, facility modernizations, or newly opened units, as these changes may alter network connectivity and predicted pathogen spread.

Fourth, several networks were presented by multiple articles, some of which relied on different subsets of the same underlying network. As a result, their reconstructed forms varied in size [5,58], in the types of facilities included [29,58], in whether indirect transfers were included [35,36], and in the time periods covered by the patient transfer data [23,87]. These differences show that, once reconstructed, a network can evolve through methodological choices to address different research questions.

Fifth, although our confidence assessment showed good overall quality, some gaps remain. The description of the underlying patient transfer database improved over time, but it was still often insufficiently described. This limited reporting can hinder the assessment of network reliability and reproducibility. Future studies should therefore provide a detailed description of the data used for network reconstruction, and, when possible, share aggregated transition matrices. Stakeholder involvement in the design and analysis processes was also limited in several articles. The relevance of this criterion should be interpreted in light of each study’s objective. It may be important for studies simulating and recommending infection prevention and control interventions, but less central for studies focused on describing patient-sharing networks.

### Limitations

Our work has several limitations. First, the articles we identified come from a wide range of disciplines, which sometimes use different terminologies to describe the same methods, potentially leading to the omission of some relevant articles. To reduce this risk, we included as many synonyms as possible in our search strategy and systematically reviewed the reference lists of all included articles. Second, we voluntarily excluded large databases giving access to grey literature (e.g., Google Scholar), to only retain original research articles in this review, although this kind of database could have provided additional networks. Third, we excluded networks constructed from data on patients with specific diseases, in order to draw conclusions about overall patient-sharing networks. However, this approach may have led us to overlook highly structured networks, such as those based on patients with carbapenem-resistant Enterobacterales [90] or with hypertension [91]. Fourth, we defined a network as “temporal” when at least one instance was observed per quarter. Although this threshold is somewhat arbitrary, it was selected to account for seasonal variations; alternative definitions might have yielded slightly different results. Fifth, when assessing pathogen transmission modelling approaches, we excluded statistical modelling articles because they did not align with the primary objective of our review. Nonetheless, such models could provide valuable insights into the transmission dynamics of pathogens in healthcare settings. Sixth, our review focused exclusively on patient transfers between healthcare facilities or units. However, hospitals collaborate in other ways as well [92]. Studies have investigated collaborations through the mobility of healthcare professionals across institutions [59] or the sharing of patients among physicians [60]. Within-ward networks where nodes represent hospital rooms have also been proposed to better understand the patterns of patient movements and staff within hospitals [61]. Seventh, the confidence assessment was performed by a single reviewer (EH), therefore inter-rater reliability could not be calculated.

### Perspectives and future directions

Future work should expand healthcare network reconstruction to include a broader range of facility types, and reconstruct more temporal networks, particularly when these networks are used in parallel to pathogen transmission models. Pathogen transmission models could focus on emerging threats including Carbapenem-Resistant Enterobacterales and *Candida auris*. Future studies could also reconstruct disease-specific patient transfer networks. For example, networks focused on patients with acute myocardial infarction could help assess whether referral pathways support timely access to centres able to perform percutaneous coronary intervention. In addition, future studies should clearly describe the patient transfer data used for network reconstruction, including their source, coverage, study period, and potential missing data. When possible, sharing aggregated transition matrices would improve reproducibility, comparability across studies and support open science. Although artificial intelligence frameworks were not used in the included studies, machine learning approaches may in the future provide complementary tools to link healthcare facility characteristics, network features, and epidemiological data. Finally, to date, few studies have attempted to integrate genomic data into patient-sharing networks. Such integration could reveal clusters of facilities sharing genetically close strains, and connect them to patient transfer patterns, which could provide a better understanding of pathogen transmission pathways across healthcare systems.

## Supporting information

S1 File PRISMA 2020 checklist. From: Page MJ, McKenzie JE, Bossuyt PM, Boutron I, Hoffmann TC, Mulrow CD, et al. The PRISMA 2020 statement: an updated guideline for reporting systematic reviews. BMJ. 2021;372:n71. Licensed under CC BY 4.0.
(DOCX)

S1 Table Detailed search queries for PubMed, Scopus and IEEE. (XLSX)

S2 Table QuADS criteria adapted for this review. (XLSX)

S3 Table Inter-rater reliability statistics. (XLSX)

S4 Table List of included and excluded articles. (XLSX)

S5 Table Data extracted from the included articles. (XLSX)

S6 Table List of networks and articles that presented them. (XLSX)

S7 Table Maximum delay between two consecutive hospital stays to be considered a patient transfer. (XLSX)

S8 Table Metrics used in the articles to describe the networks. (XLSX)

S9 Table Confidence assessment scores of all included articles. (XLSX)

S1 Fig Types and duration of databases in the networks. (A) Frequency of database types (B) Distribution of database duration.
(TIF)

S2 Fig Network characteristics for each network-article combination. (A) Number of facilities (B) Number of patient transfers per year. For networks presented in multiple articles, each point represents a value reported in an article.
(TIF)

S3 Fig Stratified quality analysis for articles published (A) between 2010 and 2014 (B) between 2015 and 2019 (C) between 2020 and 2025. (TIF)

## Abbreviations

HAI: Healthcare-associated infection
IEEE: Institute of Electrical and Electronics Engineers
PMSI: Programme de Médicalisation des Systèmes d’Information
QuADS: Quality Assessment for Diverse Studies
