# Supplementary material for: Reconstructing healthcare networks from patient transfer data: A systematic review
Source: PLOS Digit Health. 2026 Sep 15;5(9):e0001718. doi: 10.1371/journal.pdig.0001718 (PMC13577407; doi:10.1371/journal.pdig.0001718)
Supplement: Supplementary file 2 [file pdig.0001718.s014.docx]

**Journal Requirements:**

1. Please amend your detailed Financial Disclosure statement. This is published with the article. It must therefore be completed in full sentences and contain the exact wording you wish to be published.

i. Please clarify all sources of financial support for your study. List the grants, grant numbers, and organizations that funded your study, including funding received from your institution. Please note that suppliers of material support, including research materials, should be recognized in the Acknowledgements section rather than in the Financial Disclosure. 
ii. State the initials, alongside each funding source, of each author to receive each grant. For example: "This work was supported by the National Institutes of Health (####### to AM; ###### to CJ) and the National Science Foundation (###### to AM)."
iii. State what role the funders took in the study. If the funders had no role in your study, please state: “The funders had no role in study design, data collection and analysis, decision to publish, or preparation of the manuscript.”
iv. If any authors received a salary from any of your funders, please state which authors and which funders.

We amended our Financial Disclosure statement:

*L658-661: “This study was supported by the French state through the Agence Nationale de la Recherche (ANR 22 PAMR 0003, France 2030 to GB). The funders had no role in the design and conduct of the study; collection, management, analysis and interpretation of the data; preparation, review or approval of the manuscript; and decision to submit the manuscript for publication. No authors received a salary from the funders.”*

2. Some material included in your submission may be copyrighted. According to PLOS’s copyright policy, authors who use figures or other material (e.g., graphics, clipart, maps) from another author or copyright holder must demonstrate or obtain permission to publish this material under the Creative Commons Attribution 4.0 International (CC BY 4.0) License used by PLOS journals. Please closely review the details of PLOS’s copyright requirements here: PLOS Licenses and Copyright. If you need to request permissions from a copyright holder, you may use PLOS's Copyright Content Permission form.

Please respond directly to this email or email the journal office and provide any known details concerning your material's license terms and permissions required for reuse, even if you have not yet obtained copyright permissions or are unsure of your material's copyright compatibility.

Potential Copyright Issues:

a. Figure 3: please (a) provide a direct link to the base layer of the map (i.e., the country or region border shape) and ensure this is also included in the figure legend; and (b) provide a link to the terms of use / license information for the base layer image or shapefile. We cannot publish proprietary or copyrighted maps (e.g. Google Maps, Mapquest) and the terms of use for your map base layer must be compatible with our CC-BY 4.0 license.

Note: if you created the map in a software program like R or ArcGIS, please locate and indicate the source of the basemap shapefile onto which data has been plotted.

If your map was obtained from a copyrighted source please amend the figure so that the base map used is from an openly available source. Alternatively, please provide explicit written permission from the copyright holder granting you the right to publish the material under our CC-BY 4.0 license.

Please note that the following CC BY licenses are compatible with PLOS license: CC BY 4.0, CC BY 2.0 and CC BY 3.0, meanwhile such licenses as CC BY-ND 3.0 and others are not compatible due to additional restrictions.

If you are unsure whether you can use a map or not, please do reach out and we will be able to help you. The following websites are good examples of where you can source open access or public domain maps: 
* U.S. Geological Survey (USGS) - All maps are in the public domain. (<http://www.usgs.gov>) 
* PlaniGlobe - All maps are published under a Creative Commons license so please cite “PlaniGlobe, <http://www.planiglobe.com>, CC BY 2.0” in the image credit after the caption. (<http://www.planiglobe.com/?lang=enl>) 
* Natural Earth - All maps are public domain. (<http://www.naturalearthdata.com/about/terms-of-use/>)

The original version of Figure 3 used map base layers for which the terms of use were unclear. We therefore replaced all map base layers with Natural Earth map data, accessed through the rnaturalearth R package. We also revised the Figure 3 legend to explicitly report the source of the base layer and its license:

*L190-193: “Figure 3: Scale and geographic distribution of the 50 networks. (A) Number of networks by scale (B) European networks (C) North American networks. Numbers in parentheses indicate how many articles reported each network if more than one. Figure created using the Natural Earth base layer from www.naturalearthdata.com under the PDDL license.”*

3. We have noticed that you have uploaded Supporting Information files, but you have not included a list of legends. Please add a full list of legends for your Supporting Information files after the references list.

As requested, we have added a complete list of legends for all Supporting Information files after the references list.

*L736-751: “Supporting Information*

*Table S1: PRISMA checklist*

*Table S2: Detailed search queries for PubMed, Scopus and IEEE*

*Table S3: QuADS criteria adapted for this review*

*Table S4: Inter-rater reliability statistics*

*Table S5: List of included and excluded articles*

*Table S6: Data extracted from the included articles (extracted_data.xlsx)*

*Table S7: List of networks and articles that presented them*

*Table S8: Maximum delay between two consecutive hospital stays to be considered a patient transfer.*

*Table S9: Metrics used in the articles to describe the networks*

*Table S10: Confidence assessment scores of all included articles*

*Figure S1: Types and duration of databases in the networks*

*Figure S2: Network characteristics for each network-article combination*

*Figure S3: Stratified quality analysis for articles published (A) between 2010 and 2014 (B) between 2015 and 2019 (C) between 2020 and 2025.”*

4. As required by our policy on Data Availability, please ensure your manuscript or supplementary information includes the following:

A numbered table of all studies identified in the literature search, including those that were excluded from the analyses.  For every excluded study, the table should list the reason(s) for exclusion. If any of the included studies are unpublished, include a link (URL) to the primary source or detailed information about how the content can be accessed.

As requested, we have added Table S5, which provides a numbered list of all included and excluded articles at the full-text screening stage. For each excluded article, the reason for exclusion is reported. No unpublished studies were included in the review.

A table of all data extracted from the primary research sources for the systematic review and/or meta-analysis. The table must include the following information for each study:

- Name of data extractors and date of data extraction
- Confirmation that the study was eligible to be included in the review.
- All data extracted from each study for the reported systematic review and/or meta-analysis that would be needed to replicate your analyses.

As requested, we have added Table S6, which includes all data extracted from each study we included in our review, including the variables required to replicate our analyses. The table also reports the name of data extractor, date of data extraction and confirmation that each study met the eligibility criteria.

If data or supporting information were obtained from another source (e.g. correspondence with the author of the original research article), please provide the source of data and dates on which the data/information were obtained by your research group.

No data or supporting information were obtained from other sources.

If applicable for your analysis, a table showing the completed risk of bias and quality/certainty assessments for each study or outcome.  Please ensure this is provided for each domain or parameter assessed. For example, if you used the Cochrane risk-of-bias tool for randomized trials, provide answers to each of the signalling questions for each study. If you used GRADE to assess certainty of evidence, provide judgements about each of the quality of evidence factor. This should be provided for each outcome.

As requested, we revised Table S10 to provide the completed confidence assessment for each included study. The table now reports the score assigned to each criterion.

An explanation of how missing data were handled.

We added a mention about imputation in our Methods section:

*L133-134: “When information was not reported in an included article, descriptive statistics were conducted using only articles with available data for the variable considered.”*

This information can be included in the main text, supplementary information, or relevant data repository. Please note that providing these underlying data is a requirement for publication in this journal, and if these data are not provided your manuscript might be rejected.

If the reviewer comments include a recommendation to cite specific previously published works, please review and evaluate these publications to determine whether they are relevant and should be cited. There is no requirement to cite these works unless the editor has indicated otherwise.

**Additional Editor Comments (if provided):**

[Note: HTML markup is below. Please do not edit.]

**Reviewers' Comments:**

Reviewer's Responses to Questions

**Comments to the Author**

1. Does this manuscript meet PLOS Digital Health’s [publication criteria](https://track.editorialmanager.com/CL0/https:%2F%2Fjournals.plos.org%2Fdigitalhealth%2Fs%2Fjournal-information%23loc-criteria-for-publication/1/010f019df1d72f84-f0c72fed-409c-49fd-b1f0-e6c0c51f4f9e-000000/6npawoK66QrS6suhVAHdyqVv3J5Tv-BQOr2LwwjQnIU=258)? Is the manuscript technically sound, and do the data support the conclusions? The manuscript must describe methodologically and ethically rigorous research with conclusions that are appropriately drawn based on the data presented.

Reviewer #1: Yes

Reviewer #2: Yes

Reviewer #3: Yes

Reviewer #4: Yes

Reviewer #5: Yes

2. Has the statistical analysis been performed appropriately and rigorously?

Reviewer #1: N/A

Reviewer #2: Yes

Reviewer #3: Yes

Reviewer #4: N/A

Reviewer #5: Yes

3. Have the authors made all data underlying the findings in their manuscript fully available (please refer to the Data Availability Statement at the start of the manuscript PDF file)?

The [PLOS Data policy](https://track.editorialmanager.com/CL0/https:%2F%2Fjournals.plos.org%2Fdigitalhealth%2Fs%2Fdata-availability/1/010f019df1d72f84-f0c72fed-409c-49fd-b1f0-e6c0c51f4f9e-000000/iFXK5r8nZN6Jl73LotLOFR0_DiFZ0VzSdtcuZhHL-Qo=258) requires authors to make all data underlying the findings described in their manuscript fully available without restriction, with rare exception. The data should be provided as part of the manuscript or its supporting information, or deposited to a public repository. For example, in addition to summary statistics, the data points behind means, medians and variance measures should be available. If there are restrictions on publicly sharing data—e.g. participant privacy or use of data from a third party—those must be specified.

Reviewer #1: Yes

Reviewer #2: Yes

Reviewer #3: Yes

Reviewer #4: Yes

Reviewer #5: Yes

4. Is the manuscript presented in an intelligible fashion and written in standard English?

PLOS Digital Health does not copyedit accepted manuscripts, so the language in submitted articles must be clear, correct, and unambiguous. Any typographical or grammatical errors should be corrected at revision, so please note any specific errors here.

Reviewer #1: Yes

Reviewer #2: Yes

Reviewer #3: Yes

Reviewer #4: Yes

Reviewer #5: Yes

5. Review Comments to the Author

Please use the space provided to explain your answers to the questions above. You may also include additional comments for the author, including concerns about dual publication, research ethics, or publication ethics. (Please upload your review as an attachment if it exceeds 20,000 characters)

**Reviewer #1:** The manuscript presents a well-structured PRISMA-compliant systematic review on reconstructing healthcare networks from patient transfer data, with clear objectives and a reasonably rigorous screening pipeline (5,721 → 79 studies), and provides useful synthesis of network characteristics, data sources, and epidemiological applications. However, several critical limitations reduce its impact.

We thank the reviewer for their constructive comments and positive assessment of our manuscript. We have revised the manuscript accordingly and provide point-by-point responses.

First, the methodological analysis remains largely descriptive rather than analytical; although the paper reports dominance of basic metrics (degree, density) and limited use of temporal modeling, it fails to critically benchmark or compare alternative network reconstruction strategies or modeling paradigms, which weakens its contribution beyond aggregation.

We agree with the reviewer that the initial version did not sufficiently compare network reconstruction strategies or discuss how these choices may influence modelling outcomes. This point was also raised by another reviewer. To address this concern, we expanded the Results section by adding a synthesis of findings on the influence of network structure, direct versus indirect transfers, and static versus temporal network representations on modelling outcomes.

*L260-271: “Most modeling studies concluded that pathogen spread was strongly shaped by facilities’ position within the network, with highly connected hospitals, long-term acute-care hospitals and nursing homes acting as strong contributors to regional dissemination or persistence (5,9,10,26,29,36,40,41). While multiple studies showed that high network connectivity facilitated spatial dissemination (5,9,10,26,28,29,40–44), some models suggested that increased inter-hospital transfers could dilute local prevalence, depending notably on network structure (24,28).*

*Importantly, several studies indicated that modeling outcomes were sensitive to network reconstruction methodology. Models including only direct transfers underestimated both the number of facilities reached and the predicted system-wide prevalence (6,7,29,39). In addition, using static networks could bias the model by assuming that all links were simultaneously active, whereas temporal networks preserve the order and timing of patient movements and therefore better capture transmission chains (39).”*

Second, the exclusion of disease-specific studies introduces bias, especially given that pathogen-specific transfer dynamics are central to infection modeling, leading to partial insights into transmission networks.

We agree that pathogen transmission dynamics are central to infection modelling.

However, overall patient-sharing networks are directly relevant for modelling pathogen spread, as they describe patient-sharing patterns between facilities, including movements of patients whose colonization status is unknown. This is supported by Nekkab et al. (2017), who found no significant differences in patient transfer patterns when comparing general, suspected-HAI, and confirmed-HAI networks.

In addition, the objective of this review was to synthesize studies reconstructing overall patient-sharing networks, as specified in our PROSPERO protocol. Disease-specific networks were therefore outside the scope of this review.

We nevertheless agree that disease-specific networks provide a useful complementary perspective, and therefore we added the following sentence to the Perspective section:

*L412-415: “Future studies could also reconstruct disease-specific patient transfer networks. For example, networks focused on patients with acute myocardial infarction could help assess whether referral pathways support timely access to centres able to perform percutaneous coronary intervention.”*

Third, data heterogeneity and poor reporting across included studies (e.g., missing transfer counts in ~58% of articles and limited description of underlying datasets) are acknowledged but not systematically addressed through stratified quality analysis or sensitivity discussion.

We thank the reviewer for this helpful comment. To address heterogeneity in reporting quality, we added a stratified confidence assessment by publication period and expanded the Results section accordingly.

*L296-298: “Figure S3 presents the stratified confidence assessment by publication period. Scores for the “7-Recruitment data” criterion increased from 0.33 in 2010–2014 to 0.55 in 2015–2019 and 0.60 in 2020–2025.”*

Fourth, the review lacks integration with emerging AI-driven paradigms; despite discussing network-based modeling, it overlooks how modern frameworks such as retrieval-augmented generation (RAG) could enhance clinical decision support using structured network knowledge, or how large language models are transforming healthcare analytics and interpretation pipelines.

Our review focuses on studies reconstructing and analysing patient transfer networks, rather than on artificial intelligence frameworks for clinical decision support. Retrieval-augmented generation and large language models address different tasks, mainly related to text interpretation and generation. While these approaches are increasingly relevant in healthcare analytics, they differ substantially from the network reconstruction and modelling methods considered in this review.

In addition, none of the included studies used retrieval-augmented generation or large language models. We therefore considered that adding a dedicated discussion of these paradigms would fall outside the scope of our review.

Fifth, stakeholder relevance and clinical translation remain underdeveloped, with minimal discussion on operational deployment of such networks in real-world healthcare systems. To strengthen the manuscript, the authors should position their work within contemporary AI-enabled healthcare research and explicitly reference recent studies such as “Retrieval-augmented generation (RAG) in healthcare: A comprehensive review” and “ChatGPT: transforming healthcare with AI,” which provide complementary perspectives on integrating structured medical knowledge with intelligent systems for improved clinical utility.

We thank the reviewer for this comment. We agree that stakeholder relevance and clinical translation are important considerations, particularly when patient-sharing networks are used to inform infection prevention and control interventions or operational decision-making. This point was also raised by another reviewer. We have therefore expanded the “Implications for future work” section to clarify that stakeholder involvement should be interpreted according to each study’s objective.

*L380-383: “Stakeholder involvement in the design and analysis processes was also limited in several articles. The relevance of this criterion should be interpreted in light of each study’s objective. It may be important for studies simulating and recommending infection prevention and control interventions, but less central for studies focused on describing patient-sharing networks.”*

Regarding AI-enabled healthcare research, we acknowledge that approaches such as retrieval-augmented generation and large language models are increasingly relevant for clinical decision support. However, the two suggested articles do not fall within the scope of our review, which focuses specifically on healthcare networks reconstructed from patient transfer data. None of the included studies used tools such as retrieval-augmented generation or large language models as part of their methodology, therefore, we did not reference the studies suggested by the reviewer. We added a sentence in perspectives:

*L417-420: “Although artificial intelligence frameworks were not used in the included studies, machine learning approaches may provide complementary tools to link healthcare facility characteristics, network features, and epidemiological data.”*

**Reviewer #2:** This manuscript presents a well-structured and timely systematic review on patient-sharing networks and their role in pathogen transmission across healthcare systems. The use of PRISMA guidelines and a preregistered protocol strengthens the rigor and transparency of the work. The visualizations are clear and effective, making the findings easy to follow. The review also does a good job identifying key gaps in the literature, particularly the limited inclusion of non-hospital facilities and the underuse of temporal network approaches.

We thank the reviewer for their consideration and their positive assessment of our work.

One area that could benefit from further clarification is the description and consistency of underlying patient transfer data across studies, as this may affect reproducibility and comparability.

We thank the reviewer for this comment. We agree that the description of patient transfer data is essential for assessing reproducibility and comparability of the reconstructed networks. We therefore clarified this point in the Discussion.

Added text in the review findings:

*L309–311: “Overall, the confidence assessment was good across the included studies, although the description of the underlying patient transfer data was limited in multiple studies.”*

Added text in the implications for future work:

*L375–383: “Fifth, although our confidence assessment showed good overall quality, some gaps remain. The description of the underlying patient transfer database improved over time, but it was still often insufficiently described. This limited reporting can hinder the assessment of network reliability and reproducibility. Future studies should therefore provide a detailed description of the data used for network reconstruction, and, when possible, share aggregated transition matrices. Stakeholder involvement in the design and analysis processes was also limited in several articles. The relevance of this criterion should be interpreted in light of each study’s objective. It may be important for studies simulating and recommending infection prevention and control interventions, but less central for studies focused on describing patient-sharing networks.*

Added text in the Perspectives section:

*L415–418: “In addition, future studies should clearly describe the patient transfer data used for network reconstruction, including their source, coverage, study period, and potential missing data. When possible, sharing aggregated transition matrices would improve reproducibility, comparability across studies and support open science.”*

Overall, this is a valuable contribution that provides useful insights and directions for future research.

**Reviewer #3:** This systematic review addresses the methodology used to reconstruct healthcare networks from patient transfer data. The manuscript is well-structured, follows PRISMA guidelines, and provides a clear overview of the current research landscape. The authors highlight geographic biases (focus on Europe and North America) and the frequent exclusion of long-term care facilities (like Nursing homes) that have important implications for public health policy and infection control.

We thank the reviewer for their careful reading of the manuscript and for their positive assessment.

Comments
1. Depth of Results regarding Mathematical Modeling: While the review is highly effective at documenting the "selection" process (how nodes and edges are defined), the results regarding the actual application of these networks feel somewhat underdeveloped. The manuscript notes that 35.4% of the included studies utilize mathematical models for pathogen transmission, yet the synthesis of these findings is primarily descriptive. As a reader, it is difficult to discern the "so what" of these models. For instance, do different reconstruction methods significantly change the predicted speed or reach of an outbreak? I suggest the authors expand the Results and Discussion sections to synthesize the key epidemiological conclusions or takeaways from those 28 modeling papers. Without this, the paper describes how the "tools" are built but offers limited insight into what we have learned from using them. The authors can possibly bring in more data analytic figures or descriptions from the paper, and their GitHub repository, and explain meaningfully, in a manner which is understandable to the reader only reviewing the paper.

We thank the reviewer for this important comment. We agree that the epidemiological insights derived from mathematical modeling studies were not sufficiently developed in the initial manuscript. We therefore expanded the Results section to better synthesize the main conclusions from these studies.

*L260-265: “Most modeling studies concluded that pathogen spread was strongly shaped by facilities’ position within the network, with highly connected hospitals, long-term acute-care hospitals and nursing homes acting as strong contributors to regional dissemination or persistence (5,9,10,26,29,36,40,41). While multiple studies showed that high network connectivity facilitated spatial dissemination (5,9,10,26,28,29,40–44), some models suggested that increased inter-hospital transfers could dilute local prevalence, depending notably on network structure (24,28).”*

2. Focus on "Selections" vs. Outcomes: The current Results section is heavily skewed toward the administrative and structural characteristics of network reconstruction. While these methodological "selections" (e.g., direct vs. indirect transfers) are vital, the manuscript would be more impactful if it bridged the gap between these choices and their mathematical consequences. You have demonstrated that most studies ignore a vast majority of transfers; the next logical step in the Results should be to summarize how this specific data gap impacts the accuracy of the transmission models reviewed.

We thank the reviewer for this relevant comment. We added a paragraph in the Results section summarizing the impacts of network reconstruction methods on modelling outcomes.

*L266-271: “Importantly, several studies indicated that modeling outcomes were sensitive to network reconstruction methodology. Models including only direct transfers underestimated both the number of facilities reached and the predicted system-wide prevalence (6,7,29,39). In addition, using static networks could bias the model by assuming that all links were simultaneously active, whereas temporal networks preserve the order and timing of patient movements and therefore better capture transmission chains (39).”*

3. Geographic Limitations: The authors identify a lack of data from regions outside of Europe and North America. A brief mention in the Discussion about the digital health infrastructure requirements needed to bridge this gap in LMICs would add valuable context.

We thank the reviewer for this helpful comment. We added some elements in the Discussion to clarify the requirements needed to support network-based studies in LMICs.

*L318-323: “Bridging this gap will require investments in digital health infrastructure in LMICs, including designing routine recording of electronic health records with admission and discharge data, and secure governance frameworks for data linkage and sharing. These developments are important from an equity perspective, as they would help extend network-based surveillance and modelling approaches to currently underrepresented settings.”*

The manuscript is technically sound and provides a necessary taxonomy for healthcare network reconstruction. Strengthening the synthesis of the mathematical results would elevate the paper from a methodological survey to a more comprehensive contribution to the field of digital epidemiology. I recommend minor revisions to address the balance between reconstruction methods and modeling outcomes.

**Reviewer #4:** This is a timely systematic review of 79 articles describing 50 distinct patient-sharing networks reconstructed from routine patient transfer data. The manuscript follows PRISMA 2020 guidelines, is preregistered, and addresses a clear gap in the literature. The search strategy is reproducible, the adapted QuADS tool is well-operationalized, and the discussion is balanced and actionable. The public GitHub repository is comprehensive and fully satisfies PLOS data policy requirements. The manuscript is suitable for publication following minor revisions.

We thank the reviewer for their consideration and their positive assessment of our work.

As you refine the manuscript, it would be helpful to correct the author summary, which states that "about half of the studies also developed a mathematical model of pathogen spread." The actual figure is 27/79 (34.18%), which is closer to one-third, and as the primary entry point for non-specialist readers this should be accurate.

We thank the reviewer for pointing this out. We corrected the author summary to correct this overview.

*L68-69: “About a third of the studies also developed a mathematical model of pathogen spread on these networks, enabling simulations to assess infection control strategies.”*

It would also strengthen the methods to report inter-rater reliability statistics for both the screening and QuADS quality assessment stages, or to briefly acknowledge their absence as a limitation.

We thank the reviewer for this helpful comment. We added Table S4 reporting inter-rater reliability for the screening stage, and included the corresponding text in the Results section.

*L177: “Agreement between the two reviewers was substantial, with a Cohen’s kappa of 0.74 (Table S4).”*

For the QuADS quality assessment, ratings were performed by a single reviewer; inter-rater reliability could therefore not be calculated. We acknowledge this in the limitations section:

*L405-406: “Seventh, the confidence assessment was performed by a single reviewer (EH), therefore inter-rater reliability could not be calculated.”*

The abstract would benefit from slightly more precision in two places. "Few used temporal network approaches" could usefully include the figure reported in the main text, and "overall confidence in study findings...was good" would be easier for readers to interpret if accompanied by a median QuADS score or brief score summary.

We thank the reviewer for this suggestion. We revised the abstract to provide a more precise estimate for the use of temporal network approaches:

*L44-46: “The majority were restricted to hospitals, with nursing homes and other healthcare facilities rarely integrated, and 10 (20%) used temporal network approaches.”*

We also added a brief summary of the QuADS results in the abstract:

*L42-44: “Overall confidence in study findings, assessed through a modified QuADS tool, was good (mean total score of 21/33), although precision in the description of underlying patient transfer data was often limited.”*

Overall this is a well-executed and valuable contribution to the digital health and infectious disease literature.

**Reviewer #5:** This manuscript reviews studies on metropolitan, regional, and national healthcare networks worldwide, specifically examining their coverage and structural properties. This is an important topic; a comprehensive database like this will greatly benefit researchers modeling healthcare systems, designing facility-level policies, and analyzing the spread of healthcare-associated infections. The review topic is novel and, to my knowledge, has not been performed comprehensively before.

However, I think that there are a few minor points that should be addressed prior to publication:

One of the main conclusions in the review is the lack of time-dependent (temporal) networks. While the authors classify networks as temporal if they use successive intervals of three months or less, this binary classification (static vs. temporal) feels somewhat unbalanced, as the notion of time-dependency is much broader. The review would be much more complete if the authors discussed the specific complexities of temporal networks in more detail. For instance:
- What specific granularities are most needed (e.g., monthly, weekly, daily)?
- How should simulations account for structural changes to the system itself, such as facilities or wards being temporarily closed, modernized, or newly opened?
- When input data is divided into temporal periods, network derivation must rely on scarcer data. How should researchers handle issues like assigning an indirect transfer that spans across different temporal periods?

We thank the reviewer for this insightful comment. We expanded the Discussion to clarify the role of temporal granularity, structural changes in healthcare systems, and the trade-off between temporal resolution and data sparsity.

*L352-368: “Moreover, most articles reconstructed a single static network over the full period available in their database. Temporal networks capture patient transfer fluctuations over time, including weekly or seasonal variations, such as higher patient flows in winter due to respiratory viruses (61). They may be more appropriate for studies aiming to model pathogen transmission, since purely static networks may incorrectly estimate transmission risks by assuming all transfer links exist at all times (39). However, finer temporal resolutions rely on scarser patient transfer data, which may reduce the stability of network estimates. Sliding time windows can partly address this issue by increasing the number of observations, and can also smooth daily fluctuations. Conversely, static networks may provide a global overview of patient flows when temporal analyses show stable patient-sharing patterns, as reported in some studies (16,51). The choice between static and temporal networks should therefore be guided by the research question, data resolution, and temporal stability of patient flows. For studies including indirect transfers, the temporal scale used to reconstruct the network should also be longer than the maximal allowed interval between two stays, to avoid assigning indirect transfers across incompatible time windows. Future studies should also explicitly consider structural changes in the healthcare system, such as temporary ward closures, facility modernizations, or newly opened units, as these changes may alter network connectivity and predicted pathogen spread.”*

In the "Confidence assessment" section, the authors state that "Most articles demonstrated insufficient stakeholder involvement in the design and analysis processes". I fail to see why this should be treated as a universal quality criterion, as its relevance depends entirely on the specific subject of the article under consideration. I agree that stakeholder involvement is crucial when some policy is proposed and analyzed. However, a transfer network is an objective fact; it exists independently of any stakeholder's involvement in the research. While a stakeholder may provide the data to derive the network, no further involvement is inherently necessary. It may be worth acknowledging that this criterion may not be applicable to every such research.

We thank the reviewer for this comment. We agree that stakeholder involvement should not be interpreted as a universal quality criterion for all network studies and revised the Discussion accordingly.

*L375-383: “Fifth, although our confidence assessment showed good overall quality, some gaps remain. The description of the underlying patient transfer database improved over time, but it was still often insufficiently described. This limited reporting can hinder the assessment of network reliability and reproducibility. Future studies should therefore provide a detailed description of the data used for network reconstruction, and, when possible, share aggregated transition matrices. Stakeholder involvement in the design and analysis processes was also limited in several articles. The relevance of this criterion should be interpreted in light of each study’s objective. It may be important for studies simulating and recommending infection prevention and control interventions, but less central for studies focused on describing patient-sharing networks.”*

I do not understand the numbers provided in Figure 1. The initial search yielded 5721 articles, and the authors state 2669 duplicates were removed. However, the sum of the two smaller bases (Scopus and IEEE) combined is only 2269. Does this mean some records in PubMed itself were duplicated, or is this a typo? Furthermore, the math does not align: 5721-2669 is 3052, but the flowchart lists 3049 studies screened. Please clarify.

We thank the reviewer for identifying this inconsistency. It resulted from the distinction between duplicates removed automatically by Covidence and those removed manually. Covidence removed 2,669 duplicate records, and 3 additional duplicates were removed manually, giving 2,672 duplicates removed in total and 3,049 records screened. We have revised Figure 1 accordingly.

We also note that duplicate records occasionally occur within PubMed, as acknowledged by the NCBI PubMed team ([NLM Office Hours: PubMed](https://www.nlm.nih.gov/oet/ed/pubmed/08-23_oh-pubmed.html))

Figures 4 and S1 appear to be exactly the same. I suspect the images may have been mistaken during the upload of the supplementary material.

We thank the reviewer for pointing this out. Figure S1 was incorrectly uploaded in the supplementary material. We have replaced it with the correct figure.

There is some inconsistency in the formatting of the references. For example, some articles include URLs or DOIs, while most do not. This should be made uniform within the "References" section.

We thank the reviewer for noting this inconsistency. We revised the reference list to ensure consistent formatting.
