# Supplementary material for: Reconstructing healthcare networks from patient transfer data: A systematic review
Source: PLOS Digit Health. 2026 Sep 15;5(9):e0001718. doi: 10.1371/journal.pdig.0001718 (PMC13577407; doi:10.1371/journal.pdig.0001718)
Supplement: Peer review history

# Peer review: Decision Letter 0

**DOI:** 10.1371/journal.pdig.0001718.r001

**Authors:** Louise A C Millard, Sulaf Assi

4 May 2026

Response to Reviewers Revised Manuscript with Track Changes Manuscript **Journal Requirements:**

1. Please amend your detailed Financial Disclosure statement. This is published with the article. It must therefore be completed in full sentences and contain the exact wording you wish to be published.

i. Please clarify all sources of financial support for your study. List the grants, grant numbers, and organizations that funded your study, including funding received from your institution. Please note that suppliers of material support, including research materials, should be recognized in the Acknowledgements section rather than in the Financial Disclosure.

ii. State the initials, alongside each funding source, of each author to receive each grant. For example: "This work was supported by the National Institutes of Health (####### to AM; ###### to CJ) and the National Science Foundation (###### to AM)."

iii. State what role the funders took in the study. If the funders had no role in your study, please state: “The funders had no role in study design, data collection and analysis, decision to publish, or preparation of the manuscript.”

iv. If any authors received a salary from any of your funders, please state which authors and which funders.

2. Some material included in your submission may be copyrighted. According to PLOS’s copyright policy, authors who use figures or other material (e.g., graphics, clipart, maps) from another author or copyright holder must demonstrate or obtain permission to publish this material under the Creative Commons Attribution 4.0 International (CC BY 4.0) License used by PLOS journals. Please closely review the details of PLOS’s copyright requirements here: PLOS Licenses and Copyright. If you need to request permissions from a copyright holder, you may use PLOS's Copyright Content Permission form.

Please respond directly to this email or email the journal office and provide any known details concerning your material's license terms and permissions required for reuse, even if you have not yet obtained copyright permissions or are unsure of your material's copyright compatibility.

Potential Copyright Issues:

a. Figure 3: please (a) provide a direct link to the base layer of the map (i.e., the country or region border shape) and ensure this is also included in the figure legend; and (b) provide a link to the terms of use / license information for the base layer image or shapefile. We cannot publish proprietary or copyrighted maps (e.g. Google Maps, Mapquest) and the terms of use for your map base layer must be compatible with our CC-BY 4.0 license.

Note: if you created the map in a software program like R or ArcGIS, please locate and indicate the source of the basemap shapefile onto which data has been plotted.

If your map was obtained from a copyrighted source please amend the figure so that the base map used is from an openly available source. Alternatively, please provide explicit written permission from the copyright holder granting you the right to publish the material under our CC-BY 4.0 license.

Please note that the following CC BY licenses are compatible with PLOS license: CC BY 4.0, CC BY 2.0 and CC BY 3.0, meanwhile such licenses as CC BY-ND 3.0 and others are not compatible due to additional restrictions.

If you are unsure whether you can use a map or not, please do reach out and we will be able to help you. The following websites are good examples of where you can source open access or public domain maps:

* U.S. Geological Survey (USGS) - All maps are in the public domain. (http://www.usgs.gov)

* PlaniGlobe - All maps are published under a Creative Commons license so please cite “PlaniGlobe, http://www.planiglobe.com, CC BY 2.0” in the image credit after the caption. (http://www.planiglobe.com/?lang=enl)

* Natural Earth - All maps are public domain. (http://www.naturalearthdata.com/about/terms-of-use/)

3. We have noticed that you have uploaded Supporting Information files, but you have not included a list of legends. Please add a full list of legends for your Supporting Information files after the references list.

4. As required by our policy on Data Availability, please ensure your manuscript or supplementary information includes the following:

A numbered table of all studies identified in the literature search, including those that were excluded from the analyses.

For every excluded study, the table should list the reason(s) for exclusion.

If any of the included studies are unpublished, include a link (URL) to the primary source or detailed information about how the content can be accessed.

A table of all data extracted from the primary research sources for the systematic review and/or meta-analysis. The table must include the following information for each study:

Name of data extractors and date of data extraction

Confirmation that the study was eligible to be included in the review.

All data extracted from each study for the reported systematic review and/or meta-analysis that would be needed to replicate your analyses.

If data or supporting information were obtained from another source (e.g. correspondence with the author of the original research article), please provide the source of data and dates on which the data/information were obtained by your research group.

If applicable for your analysis, a table showing the completed risk of bias and quality/certainty assessments for each study or outcome.  Please ensure this is provided for each domain or parameter assessed. For example, if you used the Cochrane risk-of-bias tool for randomized trials, provide answers to each of the signalling questions for each study. If you used GRADE to assess certainty of evidence, provide judgements about each of the quality of evidence factor. This should be provided for each outcome.

An explanation of how missing data were handled.

This information can be included in the main text, supplementary information, or relevant data repository. Please note that providing these underlying data is a requirement for publication in this journal, and if these data are not provided your manuscript might be rejected.

**Additional Editor Comments (if provided):** **Reviewers' Comments:**

**Comments to the Author**

1. Does this manuscript meet PLOS Digital Health’s publication criteria?

Reviewer #1: Yes

Reviewer #2: Yes

Reviewer #3: Yes

Reviewer #4: Yes

Reviewer #5: Yes

**********

2. Has the statistical analysis been performed appropriately and rigorously?

Reviewer #1: N/A

Reviewer #2: Yes

Reviewer #3: Yes

Reviewer #4: N/A

Reviewer #5: Yes

**********

3. Have the authors made all data underlying the findings in their manuscript fully available (please refer to the Data Availability Statement at the start of the manuscript PDF file)?

The PLOS Data policy

Reviewer #1: Yes

Reviewer #2: Yes

Reviewer #3: Yes

Reviewer #4: Yes

Reviewer #5: Yes

**********

4. Is the manuscript presented in an intelligible fashion and written in standard English?

Reviewer #1: Yes

Reviewer #2: Yes

Reviewer #3: Yes

Reviewer #4: Yes

Reviewer #5: Yes

**********

**Reviewer #1:**  The manuscript presents a well-structured PRISMA-compliant systematic review on reconstructing healthcare networks from patient transfer data, with clear objectives and a reasonably rigorous screening pipeline (5,721 → 79 studies), and provides useful synthesis of network characteristics, data sources, and epidemiological applications . However, several critical limitations reduce its impact.

First, the methodological analysis remains largely descriptive rather than analytical; although the paper reports dominance of basic metrics (degree, density) and limited use of temporal modeling, it fails to critically benchmark or compare alternative network reconstruction strategies or modeling paradigms, which weakens its contribution beyond aggregation.

Second, the exclusion of disease-specific studies introduces bias, especially given that pathogen-specific transfer dynamics are central to infection modeling, leading to partial insights into transmission networks.

Third, data heterogeneity and poor reporting across included studies (e.g., missing transfer counts in ~58% of articles and limited description of underlying datasets) are acknowledged but not systematically addressed through stratified quality analysis or sensitivity discussion.

Fourth, the review lacks integration with emerging AI-driven paradigms; despite discussing network-based modeling, it overlooks how modern frameworks such as retrieval-augmented generation (RAG) could enhance clinical decision support using structured network knowledge, or how large language models are transforming healthcare analytics and interpretation pipelines.

Fifth, stakeholder relevance and clinical translation remain underdeveloped, with minimal discussion on operational deployment of such networks in real-world healthcare systems. To strengthen the manuscript, the authors should position their work within contemporary AI-enabled healthcare research and explicitly reference recent studies such as “Retrieval-augmented generation (RAG) in healthcare: A comprehensive review” and “ChatGPT: transforming healthcare with AI,” which provide complementary perspectives on integrating structured medical knowledge with intelligent systems for improved clinical utility.

**Reviewer #2:**  This manuscript presents a well-structured and timely systematic review on patient-sharing networks and their role in pathogen transmission across healthcare systems. The use of PRISMA guidelines and a preregistered protocol strengthens the rigor and transparency of the work.

The visualizations are clear and effective, making the findings easy to follow. The review also does a good job identifying key gaps in the literature, particularly the limited inclusion of non-hospital facilities and the underuse of temporal network approaches.

One area that could benefit from further clarification is the description and consistency of underlying patient transfer data across studies, as this may affect reproducibility and comparability.

Overall, this is a valuable contribution that provides useful insights and directions for future research.

**Reviewer #3:**  This systematic review addresses the methodology used to reconstruct healthcare networks from patient transfer data. The manuscript is well-structured, follows PRISMA guidelines, and provides a clear overview of the current research landscape. The authors highlight geographic biases (focus on Europe and North America) and the frequent exclusion of long-term care facilities (like Nursing homes) that have important implications for public health policy and infection control.

Comments

1. Depth of Results regarding Mathematical Modeling: While the review is highly effective at documenting the "selection" process (how nodes and edges are defined), the results regarding the actual application of these networks feel somewhat underdeveloped. The manuscript notes that 35.4% of the included studies utilize mathematical models for pathogen transmission, yet the synthesis of these findings is primarily descriptive. As a reader, it is difficult to discern the "so what" of these models. For instance, do different reconstruction methods significantly change the predicted speed or reach of an outbreak? I suggest the authors expand the Results and Discussion sections to synthesize the key epidemiological conclusions or takeaways from those 28 modeling papers. Without this, the paper describes how the "tools" are built but offers limited insight into what we have learned from using them. The authors can possibly bring in more data analytic figures or descriptions from the paper, and their GitHub repository, and explain meaningfully, in a manner which is understandable to the reader only reviewing the paper.

2. Focus on "Selections" vs. Outcomes: The current Results section is heavily skewed toward the administrative and structural characteristics of network reconstruction. While these methodological "selections" (e.g., direct vs. indirect transfers) are vital, the manuscript would be more impactful if it bridged the gap between these choices and their mathematical consequences. You have demonstrated that most studies ignore a vast majority of transfers; the next logical step in the Results should be to summarize how this specific data gap impacts the accuracy of the transmission models reviewed.

3. Geographic Limitations: The authors identify a lack of data from regions outside of Europe and North America. A brief mention in the Discussion about the digital health infrastructure requirements needed to bridge this gap in LMICs would add valuable context.

The manuscript is technically sound and provides a necessary taxonomy for healthcare network reconstruction. Strengthening the synthesis of the mathematical results would elevate the paper from a methodological survey to a more comprehensive contribution to the field of digital epidemiology. I recommend minor revisions to address the balance between reconstruction methods and modeling outcomes.

**Reviewer #4:**  This is a timely systematic review of 79 articles describing 50 distinct patient-sharing networks reconstructed from routine patient transfer data. The manuscript follows PRISMA 2020 guidelines, is preregistered, and addresses a clear gap in the literature. The search strategy is reproducible, the adapted QuADS tool is well-operationalized, and the discussion is balanced and actionable. The public GitHub repository is comprehensive and fully satisfies PLOS data policy requirements. The manuscript is suitable for publication following minor revisions.

As you refine the manuscript, it would be helpful to correct the author summary, which states that "about half of the studies also developed a mathematical model of pathogen spread." The actual figure is 27/79 (34.18%), which is closer to one-third, and as the primary entry point for non-specialist readers this should be accurate. It would also strengthen the methods to report inter-rater reliability statistics for both the screening and QuADS quality assessment stages, or to briefly acknowledge their absence as a limitation.

The abstract would benefit from slightly more precision in two places. "Few used temporal network approaches" could usefully include the figure reported in the main text, and "overall confidence in study findings...was good" would be easier for readers to interpret if accompanied by a median QuADS score or brief score summary.

Overall this is a well-executed and valuable contribution to the digital health and infectious disease literature.

**Reviewer #5:**  This manuscript reviews studies on metropolitan, regional, and national healthcare networks worldwide, specifically examining their coverage and structural properties. This is an important topic; a comprehensive database like this will greatly benefit researchers modeling healthcare systems, designing facility-level policies, and analyzing the spread of healthcare-associated infections. The review topic is novel and, to my knowledge, has not been performed comprehensively before.

However, I think that there are a few minor points that should be addressed prior to publication:

One of the main conclusions in the review is the lack of time-dependent (temporal) networks. While the authors classify networks as temporal if they use successive intervals of three months or less, this binary classification (static vs. temporal) feels somewhat unbalanced, as the notion of time-dependency is much broader. The review would be much more complete if the authors discussed the specific complexities of temporal networks in more detail. For instance:

- What specific granularities are most needed (e.g., monthly, weekly, daily)?

- How should simulations account for structural changes to the system itself, such as facilities or wards being temporarily closed, modernized, or newly opened?

- When input data is divided into temporal periods, network derivation must rely on scarcer data. How should researchers handle issues like assigning an indirect transfer that spans across different temporal periods?

In the "Confidence assessment" section, the authors state that "Most articles demonstrated insufficient stakeholder involvement in the design and analysis processes". I fail to see why this should be treated as a universal quality criterion, as its relevance depends entirely on the specific subject of the article under consideration. I agree that stakeholder involvement is crucial when some policy is proposed and analyzed. However, a transfer network is an objective fact; it exists independently of any stakeholder's involvement in the research. While a stakeholder may provide the data to derive the network, no further involvement is inherently necessary. It may be worth acknowledging that this criterion may not be applicable to every such research.

I do not understand the numbers provided in Figure 1. The initial search yielded 5721 articles, and the authors state 2669 duplicates were removed. However, the sum of the two smaller bases (Scopus and IEEE) combined is only 2269. Does this mean some records in PubMed itself were duplicated, or is this a typo? Furthermore, the math does not align: 5721-2669 is 3052, but the flowchart lists 3049 studies screened. Please clarify.

Figures 4 and S1 appear to be exactly the same. I suspect the images may have been mistaken during the upload of the supplementary material.

There is some inconsistency in the formatting of the references. For example, some articles include URLs or DOIs, while most do not. This should be made uniform within the "References" section.

**********

what does this mean?). If published, this will include your full peer review and any attached files.

**Do you want your identity to be public for this peer review?**  If you choose “no”, your identity will remain anonymous but your review may still be made public.

For information about this choice, including consent withdrawal, please see our Privacy Policy

Reviewer #1: No

Reviewer #2: **Yes:**  Elham Nasarian

Reviewer #3: **Yes:**  Seblewengel Birku Kebebew

Reviewer #4: No

Reviewer #5: No

**********

**Figure resubmission:**

**Reproducibility:**

To enhance the reproducibility of your results, we recommend that authors of applicable studies deposit laboratory protocols in protocols.io, where a protocol can be assigned its own identifier (DOI) such that it can be cited independently in the future. Additionally, PLOS ONE offers an option to publish peer-reviewed clinical study protocols. Read more information on sharing protocols at https://plos.org/protocols?utm_medium=editorial-email&utm_source=authorletters&utm_campaign=protocols

# Author comment: Author response to Decision Letter 1

**DOI:** 10.1371/journal.pdig.0001718.r002

12 Jun 2026

Attachment Submitted filename: Response to reviewers.docx

# Peer review: Decision Letter 1

**DOI:** 10.1371/journal.pdig.0001718.r003

**Authors:** Louise A C Millard, Sulaf Assi

24 Aug 2026

Reconstructing healthcare networks from patient transfer data: a systematic review

PDIG-D-26-00344R1

Dear Ms Hodbert,

We are pleased to inform you that your manuscript 'Reconstructing healthcare networks from patient transfer data: a systematic review' has been provisionally accepted for publication in PLOS Digital Health.

Before your manuscript can be formally accepted you will need to complete some formatting changes, which you will receive in a follow-up email from a member of our team.

Please note that your manuscript will not be scheduled for publication until you have made the required changes, so a swift response is appreciated.

IMPORTANT: The editorial review process is now complete. PLOS will only permit corrections to spelling, formatting or significant scientific errors from this point onwards. Requests for major changes, or any which affect the scientific understanding of your work, will cause delays to the publication date of your manuscript.

If your institution or institutions have a press office, please notify them about your upcoming paper to help maximize its impact. If they'll be preparing press materials, please inform our press team as soon as possible -- no later than 48 hours after receiving the formal acceptance. Your manuscript will remain under strict press embargo until 2 pm Eastern Time on the date of publication. For more information, please contact digitalhealth@plos.org.

Thank you again for supporting Open Access publishing; we are looking forward to publishing your work in PLOS Digital Health.

Best regards,

Sulaf Assi, PhD

Academic Editor

PLOS Digital Health

***********************************************************

**Additional Editor Comments (if provided):**

Comments are addressed to satisfactory level.

**Reviewer Comments (if any, and for reference):**

Reviewer's Responses to Questions

**Comments to the Author**

Reviewer #3: All comments have been addressed

Reviewer #4: All comments have been addressed

Reviewer #5: All comments have been addressed

**********

publication criteria?

Reviewer #3: Yes

Reviewer #4: Yes

Reviewer #5: Yes

**********

3. Has the statistical analysis been performed appropriately and rigorously?

Reviewer #3: Yes

Reviewer #4: Yes

Reviewer #5: Yes

**********

4. Have the authors made all data underlying the findings in their manuscript fully available (please refer to the Data Availability Statement at the start of the manuscript PDF file)?

The PLOS Data policy

Reviewer #3: Yes

Reviewer #4: Yes

Reviewer #5: Yes

**********

5. Is the manuscript presented in an intelligible fashion and written in standard English?

Reviewer #3: Yes

Reviewer #4: Yes

Reviewer #5: Yes

**********

Reviewer #3: The authors have thoroughly and meticulously addressed all comments raised during the initial review round.

Reviewer #4: (No Response)

Reviewer #5: The authors responded to comments and remarks of the reviewers and improved the manuscript considerably. I recommend publication of the manuscript.

**********

what does this mean?). If published, this will include your full peer review and any attached files.

**Do you want your identity to be public for this peer review?**  If you choose “no”, your identity will remain anonymous but your review may still be made public.

For information about this choice, including consent withdrawal, please see our Privacy Policy

Reviewer #3: No

Reviewer #4: No

Reviewer #5: No

**********
